# Large Anomalous Hall and Nernst Effects in High Curie‐Temperature Iron‐Based Heusler Compounds

**DOI:** 10.1002/advs.202100782

**Published:** 2021-07-08

**Authors:** Felix Mende, Jonathan Noky, Satya N. Guin, Gerhard H. Fecher, Kaustuv Manna, Peter Adler, Walter Schnelle, Yan Sun, Chenguang Fu, Claudia Felser

**Affiliations:** ^1^ Max Planck Institute for Chemical Physics of Solids Nöthnitzer Str. 40 Dresden 01187 Germany; ^2^ Department of Physics Indian Institute of Technology Delhi Hauz Khas New Delhi 110016 India; ^3^ State Key Laboratory of Silicon Materials, and School of Materials Science and Engineering Zhejiang University Hangzhou 310027 China

**Keywords:** disorder, Heusler compounds, magnetic Weyl materials, single crystals, thermoelectrics

## Abstract

The interplay between topology and magnetism has recently sparked the frontier studies of magnetic topological materials that exhibit intriguing anomalous Hall and Nernst effects owning to the large intrinsic Berry curvature (BC). To better understand the anomalous quantum transport properties of these materials and their implications for future applications such as electronic and thermoelectric devices, it is crucial to discover more novel material platforms for performing anomalous transverse transport studies. Here, it is experimentally demonstrated that low‐cost Fe‐based Heusler compounds exhibit large anomalous Hall and Nernst effects. An anomalous Hall conductivity of 250–750 S cm^−1^ and Nernst thermopower of above 2 µV K^−1^ are observed near room temperature. The positive effect of anti‐site disorder on the anomalous Hall transport is revealed. Considering the very high Curie temperature (nearly 1000 K), larger Nernst thermopowers at high temperatures are expected owing to the existing magnetic order and the intrinsic BC. This work provides a background for developing low‐cost Fe‐based Heusler compounds as a new material platform for anomalous transport studies and applications, in particular, near and above room temperature.

## Graphical abstract

This study reports large anomalous Hall and Nernst effects in high Curie‐temperature Iron‐based Heusler compounds. The positive effect of anti‐site disorder on the anomalous Hall transport is revealed. Considering the very high Curie temperature (nearly 1000 K), larger Nernst thermopowers at high temperatures are expected in the studied compounds owing to the existing magnetic order and the intrinsic Berry curvature.

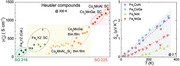

## 1 Introduction

The demand for energy‐efficient dissipationless electronics and sustainable energy conversion technologies has become a major impetus for the development of advanced materials with electronic and magnetic functionality. Topological materials are known to host exotic electronic structures that serve as an ideal platform to explore anomalous quantum transport properties for a variety of functional applications, including super‐fast electronic devices, spintronics, data storage, and thermoelectrics.^[^ ^1^, ^2^, ^3^, ^4^, ^5^ ^]^ To date, most topological phases and exotic transport behaviors have been discovered in non‐magnetic topological materials, for example, topological surface states in topological insulators,^[^ ^6^, ^7^ ^]^ ultra‐high carrier mobility,^[^ ^8^ ^]^ giant electrical and thermal magneto‐resistances in Dirac/Weyl semimetals,^[^ ^9^, ^10^, ^11^, ^12^ ^]^ and the chiral anomaly and negative magnetoresistance^[^ ^13^, ^14^ ^]^ and large Nernst effect^[^ ^15^, ^16^ ^]^ in Weyl semimetals. Magnetic topological materials,^[^ ^3^, ^17^, ^18^, ^19^, ^20^, ^21^, ^22^, ^23^, ^24^, ^25^ ^]^ in which topology and magnetism are present in the same system, provide new opportunities for the realization of the Berry‐curvature‐induced anomalous transverse transport phenomena, that is, anomalous Hall effect (AHE)^[^ ^18^ ^]^ and anomalous Nernst effect (ANE),^[^ ^19^, ^20^, ^21^ ^]^ which show potential applications in power electronics and thermoelectrics.

Very recently, two representative ferromagnetic Weyl semimetals, the Kagomé crystal Co_3_Sn_2_S_2_ and the Heusler compound Co_2_MnGa, have been identified by precise band‐structure measurements using angle‐resolved photoemission spectroscopy^[^ ^22^, ^23^ ^]^ and scanning tunneling spectroscopy.^[^ ^24^ ^]^ Benefiting from the large Berry curvature (BC) induced by the topological electronic structure,^[^ ^3^ ^]^ both compounds have shown strong AHE and ANE behavior in transverse transport. The maximum measured anomalous Hall conductivity values ($\sigma_{\mathrm{xy}}^{A}$) of Co_3_Sn_2_S_2_ and Co_2_MnGa were above 1000 S cm^−1^,^[^ ^17^, ^26^, ^27^ ^]^ whereas maximum anomalous Nernst thermopowers (*S*_xy_) of 7 µV K^−1^ for Co_2_MnGa^[^ ^19^, ^20^ ^]^ and 3 µV K^−1^ for Co_3_Sn_2_S_2_^[^ ^28^ ^]^ were obtained,^[^ ^19^, ^20^, ^28^ ^]^ approximately an order of magnitude higher than that of topologically trivial magnetic systems.^[^ ^17^, ^19^ ^]^ These encouraging results demonstrate that ferromagnetic topological materials are excellent platforms for the observation of quantum anomalous Hall states in the 2D limit^[^ ^17^ ^]^ and the realization of transverse thermoelectric conversion.^[^ ^5^, ^29^ ^]^ In addition, theoretical calculations have strengthened the understanding of electronic topology in magnetic materials. A large BC, which is closely related to the number of topological nodal lines,^[^ ^17^, ^22^, ^26^ ^]^ is thought to be the origin of the large AHE and ANE. Generally, magnetic topological materials with more mirror symmetries can host many topological nodal lines and thus, show a large BC.^[^ ^3^, ^26^ ^]^

Heusler compounds are a widely studied class of intermetallic materials with plenty of magnetic members that have high Curie temperature (*T*_c_).^[^ ^3^ ^]^ Hence, they provide a good platform for the exploration of new ferromagnets with large anomalous transverse effects.^[^ ^3^ ^]^ To date, the investigations of Heusler compounds for anomalous transport have mainly focused on Co‐based members with a *T*_c_ of approximately 694 K^[^ ^30^ ^]^ for Co_2_MnGa^[^ ^19^, ^20^, ^22^, ^26^ ^]^ and Co_2_MnAl.^[^ ^31^, ^32^, ^33^ ^]^ In contrast, low‐cost Fe‐based Heusler compounds with a much higher *T*_c_ (up to 1000 K)^[^ ^34^, ^35^ ^]^ have rarely been studied for anomalous transverse transport. Very recently, Noky et al.^[^ ^36^ ^]^ performed a comprehensive study of the intrinsic anomalous transport for magnetic cubic Heusler compounds and predicted many new Fe‐based Heusler candidates with very large AHE and ANE, which have not yet been experimentally verified.

The synthesized crystals of Fe‐based Heusler compounds tend to have a strong anti‐site disorder,^[^ ^37^, ^38^ ^]^ which is a structural defect that is generally undesirable for high‐performance unary and binary semiconductor devices.^[^ ^39^ ^]^ However, in ternary and quaternary systems, the anti‐site disorder could be used to tune the electronic structure,^[^ ^40^, ^41^ ^]^ and the electrical and magnetic properties of the crystalline materials.^[^ ^39^, ^42^ ^]^ This raises an interesting question regarding the effect of the anti‐site disorder on anomalous transverse transport in magnetic topological materials. In previous studies on Co‐based magnetic Heusler compounds, the anti‐site disorder was thought to be unfavorable for increasing the AHE: compared to the large values in well‐ordered single crystals (SC) (900–1300 S cm^−1^ at 300 K),^[^ ^22^, ^26^, ^32^ ^]^ Co_2_MnGa and Co_2_MnAl thin films with the anti‐site disorder generally had smaller $\sigma_{\mathrm{xy}}^{A}$ values (100–800 S cm^−1^ at 300 K).^[^ ^33^, ^43^, ^44^, ^45^ ^]^

This study aimed to investigate the AHE and ANE behavior of high‐*T*_c_ Fe‐based Heusler compounds Fe_2_ *YZ* (*Y* = Co, Ni; *Z* = Al, Ga).^[^ ^36^ ^]^ Although the studied Fe_2_ *YZ* SC showed strong anti‐site disorder, they showed large $\sigma_{\mathrm{xy}}^{A}\;$values (250–700 S cm^−1^) and anomalous Nernst thermopower (2 µV K^−1^ near room temperature), comparable to those of the disordered Co_2_Mn*Z* system.^[^ ^33^, ^43^, ^44^, ^45^ ^]^ With the help of theoretical calculations, we argued that the anti‐site disorder in Fe_2_ *YZ* can benefit their anomalous transport. Moreover, the anomalous Nernst thermopower of Fe_2_ *YZ* shows an approximately linear increase with rising temperature, indicating higher values could be obtained at elevated temperatures considering their high *T*_c_ values. These results demonstrate the potential of using low‐cost Fe‐based Heusler compounds for anomalous transport studies over a wide temperature range.

## 2 Results and Discussion

**Figure 1:**
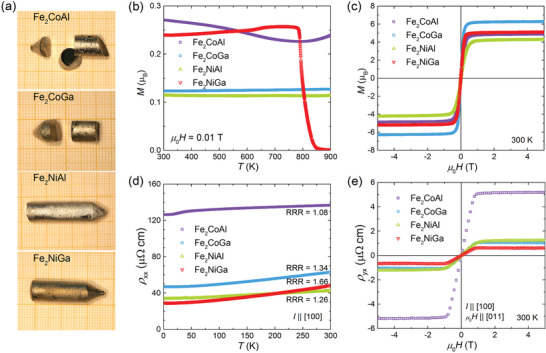
Characterization of the as‐grown Fe_2_ *YZ* SC. a) Photographs of the crystals on a 1 × 1 mm^2^ grid. b) Temperature‐dependent magnetization *M* measured at a magnetic field of 0.01 T. c) Magnetic‐field‐dependent *M* per formula unit at 300 K. d) Temperature‐dependent longitudinal resistivity *ρ*_xx_. RRR = *ρ*_xx_(300 K)/*ρ*_xx_(2 K). e) Hall resistivity *ρ*_yx_ versus magnetic field at 300 K.

SC of Fe_2_ *YZ* were grown using the Bridgman method (as described in the Experimental Section). As shown in **Figure** **1**a, the as‐grown crystals had a metallic luster. The crystallinity and orientation of the crystals were investigated using white‐beam backscattering Laue X‐ray diffraction (XRD) at room temperature, which showed distinct diffraction spots (Figure S1, Supporting Information). The polarized light and backscattered electron microscopy images (Figures S2 and S3, Supporting Information) indicated the chemical homogeneity of the grown crystals. The composition was examined using wavelength‐dispersive X‐ray spectroscopy (WDX) and inductively coupled plasma‐optical emission spectroscopy (ICP‐OES) analyses. As summarized in Tables S1 and S2, Supporting Information, the actual compositions of Fe_2_CoAl, Fe_2_NiAl, and Fe_2_NiGa SC were close to the nominally designed ones, whereas the Fe_2_CoGa SC deviated from the nominal composition with an excess of Fe and deficiency of Ga.

For performing the magnetic and transport properties measurements, the SC were first cut into bars with the longest and the two shorter sides along the [100] and [011] directions, respectively. First, the magnetic properties of the as‐grown SC were evaluated. To obtain the *T*_c_ values of the crystals, *M*‐*T* measurements were performed at a small magnetic field of 0.01 T. As shown in Figure 1b, the *M*‐*T* curves of Fe_2_CoAl, Fe_2_CoGa, and Fe_2_NiAl SC did not show a decrease with increasing temperature until 900 K, suggesting that they had *T*_c_ values above 900 K. Fe*_2_*NiGa had the lowest *T*_c_ among the studied SC, but still reached a value above 800 K, higher than that of Co_2_MnGa and Co_2_MnAl (around 694 K).^[^ ^24^, ^26^, ^30^ ^]^ These magnetic properties of the Fe_2_ *YZ* SC studied here are consistent with previous studies of polycrystalline samples, which reported *T*_c_ values of 990 K (Fe_2_CoAl),^[^ ^34^ ^]^ 1165 K (Fe_2_CoGa),^[^ ^35^ ^]^ 1010 K (Fe_2_NiAl),^[^ ^34^ ^]^ and 845 K (Fe_2_NiGa).^[^ ^38^ ^]^ Figure 1c shows the *M*‐*H* hysteresis loops for the four SC at 300 K. The crystals showed soft magnetic behavior and large saturation magnetizations with a maximum *M*_S_ of 6.27 *μ*_B_ found for Fe_2_CoGa. The saturation magnetization increased with decreasing temperature for all crystals. A maximum *M*_S_ of 6.38 *μ*_B_ at 2 K was obtained for Fe_2_CoGa (Figure S4a, Supporting Information).

**Figure 2:**
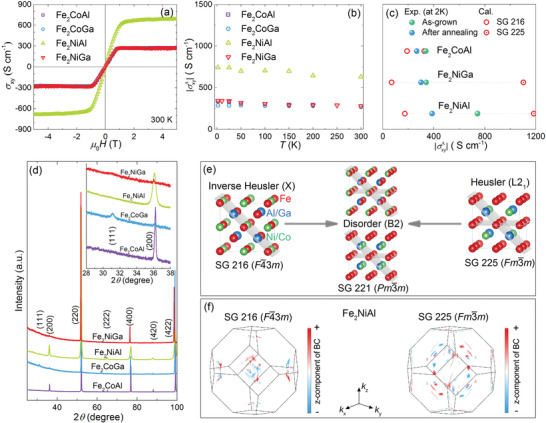
Anomalous Hall transport properties in Fe_2_ *YZ*. a) Magnetic‐field‐dependent Hall conductivity *σ*_xy_ at 300 K. b) Temperature‐dependent anomalous Hall conductivity $\sigma_{\mathrm{xy}}^{A}$. c) A comparison of the calculated $\sigma_{\mathrm{xy}}^{A}$ and experimental data. The calculations were performed based on two ordered structures, that is, the Heusler structure (L2_1_) and the inverse Heusler structure (X) shown in (e). d) Powder XRD patterns for the as‐grown SC. The inset shows the magnified pattern in the range of 28° ≤ 2*θ* ≤ 38°. e) Crystal structures of Heusler compounds with the X, L2_1_, and B2‐type structures. The grey plane indicates the mirror plane in the structure. f) BC distribution in the Brillouin zone of Fe_2_NiAl assuming it crystallizes in the inverse Heusler and normal Heusler structures.

After confirming that the fabricated Fe_2_ *YZ* SC were ferromagnetic with high *T*_c_, their transport properties were measured. The temperature‐dependent longitudinal resistivity *ρ*_xx_ of the as‐grown Fe_2_ *YZ* SC showed metallic transport behavior (Figure 1d). All crystals show the residual‐resistivity ratio (RRR) near 1, which is attributed to strong carrier scattering due to the anti‐site disorder. The magneto‐resistivity of the Fe_2_ *YZ* SC was also measured, which showed a very weak dependence on the magnetic field, even at 2 K (Figure S4b, Supporting Information). The magnetic field dependence of the Hall resistivity *ρ*_yx_ at 300 K is shown in Figure 1e. A fast change in *ρ*_yx_ at low magnetic fields (below 1 T) was observed. For magnetic fields above 1 T, *ρ*_yx_ was almost constant, suggesting that the anomalous component reached saturation. With decreasing temperature down to 2 K, the anomalous component of *ρ*_yx_ showed a slight decrease for Fe_2_CoGa, Fe_2_NiAl, and Fe_2_NiGa but remained almost unchanged for Fe_2_CoAl (Figure S4c, Supporting Information). With the measured magnetic‐field‐dependent *ρ*_xx_ and *ρ*_yx_, the Hall conductivity *σ*_xy_ can be derived using: *σ*_xy_ = *ρ*_yx_/($\rho_{yx}^{2}$ + $\rho_{xx}^{2}$). As shown in **Figure** **2**a, the *σ*_xy_ values of Fe_2_CoAl, Fe_2_CoGa, and Fe_2_NiGa were similar at 300 K, while Fe_2_NiAl had a value almost double that of the other samples above 1 T. At 2 K, the magnetic‐field‐dependent *σ*_xy_ was similar to that at 300 K (Figure S4d, Supporting Information). Using these data, $\sigma_{xy}^{A}$ was estimated by interpolating the high‐field *σ*_xy_ data to the *μ*_0_ *H* → 0 value. The estimated $\sigma_{xy}^{A}$ for Fe_2_ *YZ* shown in Figure 2b had high values of 250–750 S cm^−1^ in the temperature range of 2 to 300 K, which are smaller than those of ordered Co_2_Mn*Z* SC,^[^ ^22^, ^26^, ^32^, ^46^ ^]^ but comparable to those obtained for the corresponding thin films.^[^ ^33^, ^43^, ^44^, ^45^ ^]^ Moreover, the $\sigma_{\mathrm{xy}}^{A}$ showed a very weak temperature dependence (Figure 2b), which together with the *σ*_xx_‐independence of $\sigma_{\mathrm{xy}}^{A}$ (Figure S5, Supporting Information) suggest that the $\sigma_{\mathrm{xy}}^{A}$ of Fe_2_ *YZ* is mostly a result of the intrinsic contribution, that is, the BC.

To understand the underlying anomalous transport mechanism, we performed density‐functional theory (DFT) calculations on Fe_2_ *YZ* (see the Experimental Section for details). Generally, Heusler compounds can crystallize in two types of ordered structures, that is, the normal Heusler structure and inverse Heusler structure, which belong to the space groups *Fm* $\bar{3}$ *m* (SG 225) and *F* $\bar{4}$3*m* (SG 216), respectively. These normal and inverse structures are often denoted as L2_1_ and X, respectively, in the literature using the Strukturberichte notations.^[^ ^47^ ^]^ According to previous studies,^[^ ^37^, ^38^ ^]^ the X structure has lower total energy than the L2_1_ structure for Fe_2_ *YZ*. To compare these hypothetical structures, we performed DFT calculations for Fe_2_ *YZ*, where the band structure, density of states (DOS), $\sigma_{\mathrm{xy}}^{A}$, and anomalous Nernst conductivity $\alpha_{\mathrm{yx}}^{A}$ are presented in Figures S6–S13, Supporting Information. The calculated band structures and DOS demonstrate the metallic behavior and the co‐existence of the complex non‐topological bands and linearly crossed topological bands near the Fermi level of Fe_2_ *YZ*. As for the L2_1_ structure, the band inversion forms nodal line band structures in the *m*_x_, *m*_y_, and *m*_z_ planes in the condition without the consideration of spin‐orbital coupling (SOC), such nodal lines can be broken by the combination of SOC and the applied magnetic field. In our measurement, the applied magnetic field is along [011] direction. It can break all the three mirror symmetries and form band anti‐crossings in *k*_x_ = 0, *k*_y_ = 0, and *k*_z_ = 0 planes. The effective overlap between Fermi level and the anti‐crossing loops contributes to the intrinsic AHE and ANE. Since the three mirror planes of *m*_x_, *m*_y_, and *m*_z_ are absent in the X structure, such kind of nodal lines don't exist in them. For comparison, the calculated and experimental values are shown together in Figure 2c. Interestingly, the calculated $\sigma_{\mathrm{xy}}^{A}$ values for the X structure are smaller than those for the L2_1_ structure, while the experimental data are located in between the two calculated values.

This inconsistency between the experimental and calculated data probably arises due to the fact that the as‐grown Fe_2_ *YZ* SC neither crystallizes in the X nor L2_1_ structure. Figure 2d shows the powder XRD patterns of the as‐grown SC. One distinct feature is that the (111) diffraction peak, which is a characteristic peak for the X and L2_1_ structures, was not present for Fe_2_CoAl, Fe_2_NiAl, and Fe_2_NiGa (the inset in Figure 2d shows magnified XRD patterns). The lack of the (111) diffraction peak in Heusler compounds usually indicates the existence of a B2‐type disorder (see detailed analysis in Table S3, Supporting Information).^[^ ^47^ ^]^ In addition, the measured ^57^Fe Mössbauer spectra suggested strong disorder in the SC (Figure S14, Supporting Information). The B2‐type disorder was also previously reported for polycrystalline Fe_2_ *YZ*.^[^ ^38^, ^48^, ^49^ ^]^ Hence, the B2‐type disorder commonly occurs in Fe_2_ *YZ* crystals, regardless of their crystallinity and synthesis method. For clarity, the evolution from the X and L2_1_ structures to the B2‐type one is illustrated in Figure 2e. There could be two types of B2 structure: the B2a structure derived from the X structure with a mixture of Fe/*Y* in the Wyckoff sites (4a and 4b) and Fe/*Z* (4c and 4d sites); and the other B2b‐type derived from the L2_1_ structure with a mixture of *Y*/*Z* at the 4c and 4d sites, while both Fe atoms are located at the 4a and 4b sites. The B2‐type Heusler compounds crystallize in the space group *Pm* $\bar{3}$ *m* (SG 221). Compared to the X structure (*F* $\bar{4}$3*m*), the B2‐type (*Pm* $\bar{3}$ *m*) and the L2_1_ (*Fm* $\bar{3}$ *m*) structures have more mirror planes (grey planes in Figure 2e). The previous theoretical calculations suggest that more mirror planes result in more nodal lines and a larger BC, which is responsible for the high $\sigma_{\mathrm{xy}}^{A}$ in magnetic Heusler compounds with the L2_1_ structure.^[^ ^26^, ^36^ ^]^ It is worth noting that a small (111) peak is still observed for the studied Fe_2_CoGa SC (Figure 2d), which indicates this sample might not exhibit a strong B2‐type disorder as the other three. This was thought to be related to its actual composition that is Fe_2.19_Co_0.99_Ga_0.82_ according to the WDX result (Table S1, Supporting Information). Then the comparison between the experimental data and the calculated ones (using the composition Fe_2_CoGa) might not be reasonable and thus not shown in Figure 2c.

In this study, the hypothetical ordered ground structure of Fe_2_ *YZ* was the X structure,^[^ ^37^, ^38^ ^]^ which has fewer mirror planes than the L2_1_ structure and thus, a smaller BC (Figure 2f) and calculated $\sigma_{\mathrm{xy}}^{A}$ (Figure 2c). Furthermore, the as‐grown Fe_2_ *YZ* SC showed a strong B2‐type disorder, which could increase the number of mirror planes and thus enhance the BC. As a result, higher $\sigma_{\mathrm{xy}}^{A}$ values were observed for the as‐grown SC compared to the calculated value based on the hypothetical X structure (Figure 2c). To further confirm this, the as‐measured SC were annealed for 14–21 days below the order‐disorder transition temperature (identified by DSC analysis; Figures S15 and S16, Supporting Information) in an attempt to promote the transformation of the crystals into the ordered X structure. Powder XRD for the annealed crystals was performed (Figure S17, Supporting Information), of which a small (111) peak was observed for the annealed Fe_2_NiGa, indicating a possible promotion to the ordered X structure. In addition, a split of the diffraction peak at high angles was observed for Fe_2_NiAl, suggesting the existence of phase separation after the annealing. Then, the $\sigma_{\mathrm{xy}}^{A}$ values of the annealed Fe_2_ *YZ* crystals were measured. As shown in Figure 2c, a slight reduction in $\sigma_{\mathrm{xy}}^{A}$ was observed after annealing (Figure 2c). This may indicate a smaller BC, as the more ordered Fe_2_ *YZ* crystals with the X structure have fewer mirror planes. Conversely, the higher $\sigma_{\mathrm{xy}}^{A}$ values of the as‐grown Fe_2_ *YZ* SC could originate from the strong anti‐site‐disorder‐induced enhancement of the BC, as the B2‐type structure could have more mirror planes. This is in contrast to the Co_2_Mn*Z* Heusler compounds, whose ground‐state structure is the L2_1_ structure and the B2‐type disorder seems to suppress the $\sigma_{\mathrm{xy}}^{A}$.^[^ ^33^ ^]^ It is worth noting that the sketch for the “B2 type structure” (Figure 2e) displays averaged electron densities on the corresponding positions. This would imply a high symmetry for this crystal. But this is only an effective symmetry seen on average over the disorder over a large number of unit cells. Whereas, a completely ordered L2_1_‐type structure has these true mirror symmetries even on the scale of the conventional unit cell. That might explain why Co_2_MnZ compounds with the B2‐type disorder show decreased $\sigma_{\mathrm{xy}}^{A}$.^[^ ^33^ ^]^

**Figure 3:**
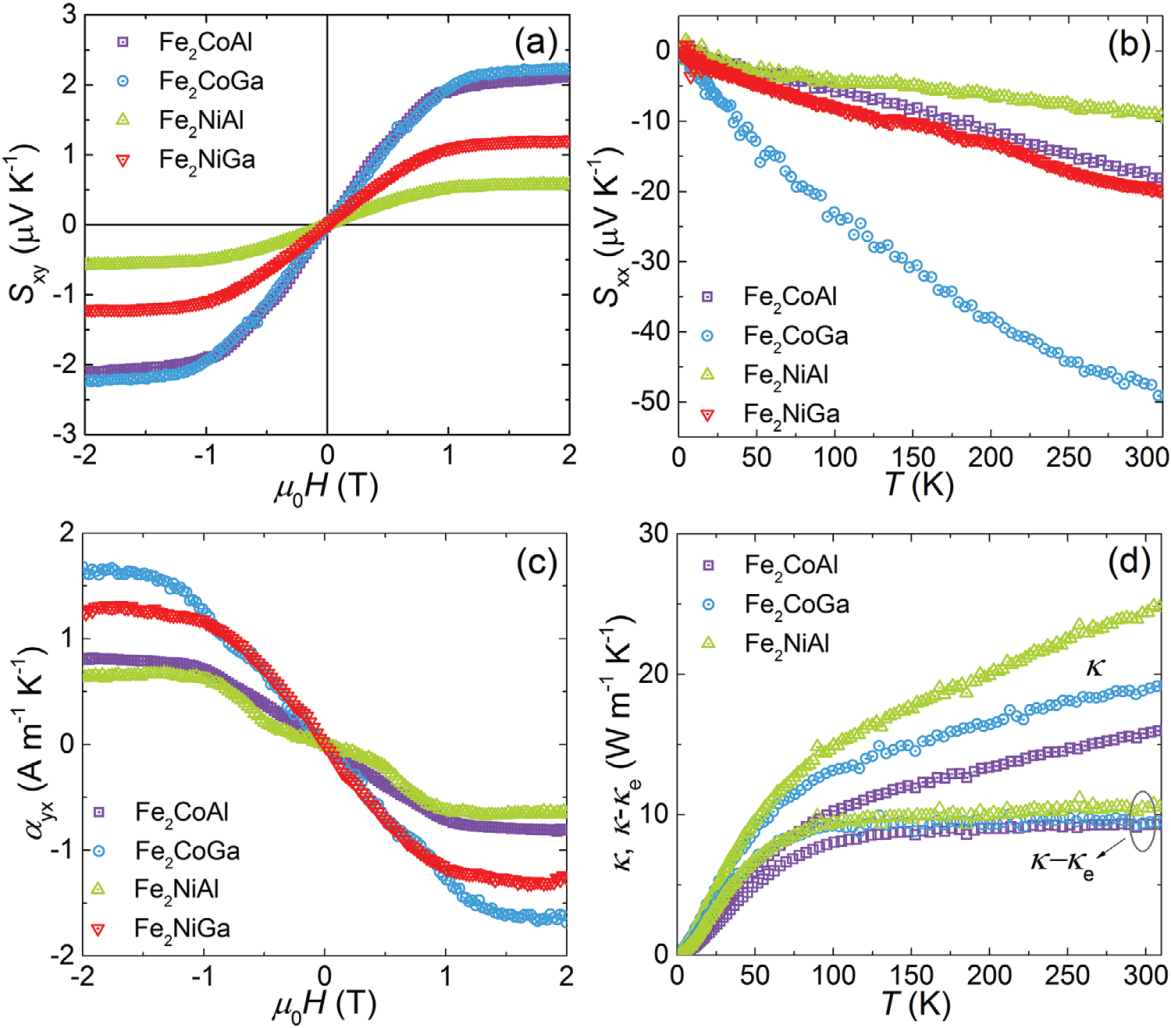
a) Magnetic field dependence of the Nernst thermopower *S*_xy_ for Fe_2_ *YZ* near 340 K, b) Temperature dependence of the Seebeck coefficient *S*_xx_, c) The estimated transverse thermoelectric conductivity *α*_yx_ near room temperature, d) Temperature dependence of thermal conductivity *κ* at 0 T.

The large BC in magnetic topological materials could generate an appreciable ANE in addition to a strong AHE, as demonstrated in Co_2_MnGa^[^ ^19^, ^20^ ^]^ and Co_3_Sn_2_S_2_.^[^ ^28^ ^]^ **Figure** **3**a shows the magnetic‐field‐dependent Nernst thermopower *S*_xy_ for the four Fe_2_ *YZ* SC measured near room temperature. Above 1 T, *S*_xy_ reaches a saturated value, with a maximum value of above 2 µV K^−1^ for Fe_2_CoAl and Fe_2_CoGa. Although these values are smaller than that of the ordered Co_2_Mn*Z* SC, they are comparable to those obtained in disordered Co_2_Mn*Z* thin films.^[^ ^33^, ^44^ ^]^ Figure 3b presents the Seebeck coefficient *S*_xx_ of Fe_2_ *YZ* SC measured without an applied magnetic field, which showed an almost linear increase with temperature, typical for a metallic system. It is worth noting that *S*_xx_ does not show an obvious change with the applied magnetic field, similar to the magnetic‐field‐independent behavior of *ρ*_xx_. The anomalous transverse thermoelectric conductivity *α*_yx_ can be estimated using the measured longitudinal and Hall resistivities (*ρ*_xx_ and *ρ*_yx_), and Nernst and Seebeck thermopowers (*S*_xy_, S_xx_), that is, *α*_yx_ = (*S*_yx_ *ρ*_xx –_ *S*_xx_ *ρ*_yx_)/(*ρ*_xx_^2^ + *ρ*_yx_^2^). Figure 3c shows the calculated *α*_yx_ near room temperature. The anomalous component $\alpha_{\mathrm{yx}}^{A}$ of all four Fe_2_ *YZ* SC reached values above 0.5 A m^−1^ K^−1^, with a maximum value of 1.7 A m^−1^ K^−1^, which is approaching the maximum value obtained in the representative magnetic Weyl systems Co_3_Sn_2_S_2_ and Co_2_Mn*Z*.^[^ ^28^, ^33^ ^]^ In addition to the thermoelectric transport properties, the thermal conductivity *κ* for as‐grown Fe_2_CoAl, Fe_2_CoGa, and Fe_2_NiAl is presented in Figure 3d. Besides, the *κ* for Fe_2_NiGa after annealing is shown in Figure S18, Supporting Information. Near room temperature, Fe_2_ *YZ* showed a moderate *κ* of about 20 W m^−1^ K^−1^. Using the Wiedemann–Franz law, the electronic thermal conductivity was calculated as *κ*_e_ = *L*_0_ *T*/*ρ*_xx_, where *L*_0_ is the constant Lorenz number and is equal to 2.44 × 10^−8^ W Ω K^−2^. As shown in Figure 3d, the other component *κ*‐*κ*_e_, which is mainly contributed by the phonons, had similar values for Fe_2_ *YZ* over the whole studied temperature range. It is worth noting that the ordered Co_2_MnGa SC shows the phonon‐phonon Umklapp scattering (indicated by a *T* ^−1^ dependence) dominated lattice thermal conductivity near 300 K,^[^ ^20^ ^]^ but such a phenomenon is not observed in the studied Fe_2_ *YZ* crystals, which could be the result of the strong disorder that induces strong point defect scattering of phonons.

**Figure 4:**
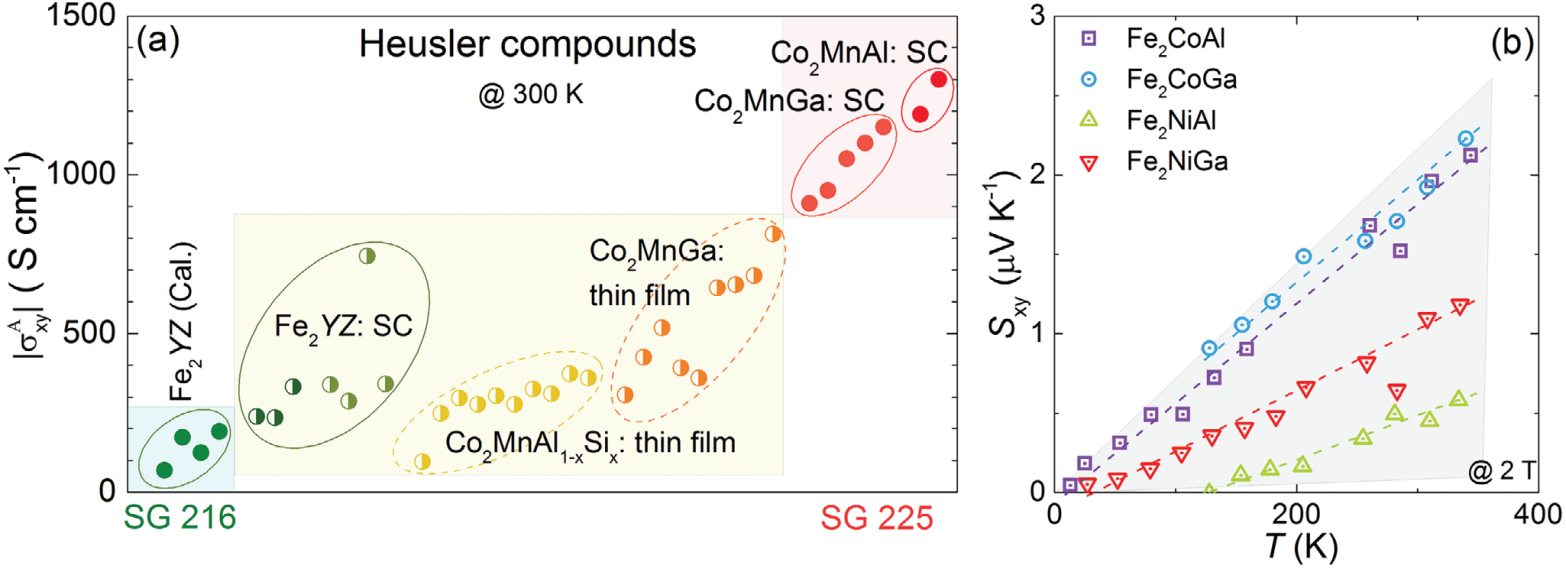
a) Anomalous Hall conductivity $|\sigma_{\mathrm{xy}}^{A}$| measured at 300 K for Fe_2_ *YZ*, Co_2_MnGa^[^ ^19^, ^22^, ^46^ ^]^ and Co_2_MnAl^[^ ^32^ ^]^ SC, and Co_2_MnGa^[^ ^43^, ^44^, ^45^ ^]^ and Co_2_Mn_1‐_ *_x_*Si*_x_* ^[^ ^33^ ^]^ thin films. For comparison, the calculated $\sigma_{\mathrm{xy}}^{A}$ for Fe_2_ *YZ* materials based on the X structure are also shown (denoted as Cal.). b) Temperature‐dependent Nernst thermopower *S*_xy_ for Fe_2_ *YZ* SC at 2 T. The dashed lines indicate the approximately linear increase of *S*_xy_ with increasing temperature.

As an extended discussion, it is meaningful to compare the anomalous transverse transport properties of Fe_2_ *YZ* and Co_2_Mn*Z*, as the latter are the Heusler compounds with the largest AHE and ANE reported to date. **Figure** **4**a shows a summary of $\sigma_{\mathrm{xy}}^{A}$ values for a range of Heusler compounds as a function of their structure. Co_2_Mn*Z* SC with the L2_1_ structure show the largest $\sigma_{\mathrm{xy}}^{A}$, which is consistent with their large number of nodal lines and thus strong BC close to *E*_F_.^[^ ^26^, ^36^ ^]^ In Co_2_Mn*Z* thin films, B2‐type disorder was found, which suppresses the $\sigma_{\mathrm{xy}}^{A}$.^[^ ^33^ ^]^ In contrast, hypothetical ordered Fe_2_ *YZ* is expected to be crystallized in the X structure and exhibits a lower $\sigma_{\mathrm{xy}}^{A}$ due to the smaller BC (Figure 2c). However, a strong B2‐type disordered structure seems to be energetically favored and therefore experimentally observed. This B2‐type structure could somewhat increase the mirror planes in the fabricated SC (Figure 2e) and enhance the BC. As a result, the $\sigma_{\mathrm{xy}}^{A}\;$of Fe_2_ *YZ* SC reaches the values of Co_2_Mn*Z* thin films (Figure 4a). This indicates that anti‐site disorder could help enhance anomalous transverse transport in Heusler compounds with the ground state structure of *F* $\bar{4}$3*m*. Moreover, since the single crystals of Fe_2_ *YZ* have already exhibited a strong anti‐site disorder, their thin films could have a similar level of anti‐site disorder and thus show a similar $\sigma_{\mathrm{xy}}^{A}$.

The temperature‐dependent Nernst thermopower is presented in Figure 4b. For all four Fe_2_ *YZ* crystals, *S*_xy_ increased almost linearly with increasing temperature. Considering that the intrinsic Berry‐curvature‐induced anomalous transport phenomena will be present while magnetic order exists in the magnetic topological materials, it can be expected that the *S*_xy_ of Fe_2_ *YZ* will continue to increase with increasing temperature until around the *T*_c_. Since the *T*_c_ of Fe_2_ *YZ* is much higher than those of Co_2_Mn*Z*, the former could thus show advantages for high‐temperature thermoelectric energy conversion based on the ANE. In a very recent study, Li et al.^[^ ^50^ ^]^ built a new monomaterial Nernst thermopile using the antiferromagnet Mn_3_Sn, suggesting a potential way for a new generation of thermopiles. It is worth noting that owing to the higher Curie temperature and larger Nernst thermopower, Fe_2_ *YZ* compounds could be good candidates for new Nernst thermopiles studies.

## 3 Conclusion

A comprehensive study of the anomalous transverse transport properties of four selected Fe‐based Heusler SC was performed. Large values of 250–750 S cm^−1^ and *S*_xy_ values of above 2 µV K^−1^ were observed near room temperature. The almost temperature‐independent $\sigma_{\mathrm{xy}}^{A}$ values indicated that the anomalous transport originates from the intrinsic BC. The anti‐site disorder and its effect on BC and $\sigma_{\mathrm{xy}}^{A}$ were discussed in relation to theoretical calculations. The anti‐site disorder in Fe‐based Heusler compounds might help to induce more mirror planes in the system and thus enhance the Berry‐curvature‐induced anomalous transport behavior. There are two conclusions from the current work which might inspire future studies: 1) Fe_2_ *YZ* thin films, which could have a similar anti‐site disorder as their single‐crystal counterparts, might exhibit similarly large anomalous transverse transport behavior; 2) The high *T*_c_ of Fe_2_ *YZ* compounds guarantees a further increase in the anomalous Nernst thermopower at elevated temperatures. These results highlight low‐cost Fe‐based Heusler compounds as a new platform for anomalous Hall and Nernst transport studies with potential applications for future electronics and thermoelectrics.

## 4 Experimental Section

### Single Crystal Growth and Characterization

SC of Fe_2_NiAl, Fe_2_CoAl, Fe_2_NiGa, and Fe_2_CoGa were grown using the Bridgman–Stockbarger crystal growth technique. First, 10 g of high‐purity elements (>99.9%) in a stoichiometric ratio were reacted using an arc melter under an argon gas atmosphere; this melting process was repeated 5 times. The as‐cast ingots were turned over after each melting process. Additionally, a Ti sponge was used as an oxygen scavenger to minimize oxygen contamination of the ingot. All ingots were subsequently sealed in quartz ampoules under argon gas atmosphere and further heat‐treated (Fe_2_NiAl: 900 °C for 4 days;^[^ ^48^ ^]^ Fe_2_CoAl: 600 °C for 14 days;^[^ ^51^ ^]^ Fe_2_NiGa: 800 °C for 14 days;^[^ ^52^ ^]^ and Fe_2_CoGa: 900 °C for 10 days^[^ ^53^ ^]^) and then quenched in ice water. Then, the annealed ingots were crushed and packed into a custom sharp‐edged alumina tube (10 mm in inner diameter), which was sealed in a tantalum tube under an argon atmosphere (0.2 atm). The compound's melting point was determined using differential scanning calorimetry (DSC 404, NETZSCH) measurements (Figure S15, Supporting Information). The as‐sealed tantalum tubes were heated to 1500 °C for Fe_2_NiAl, 1550 °C for Fe_2_CoAl, 1400 °C for Fe_2_NiGa, and 1380 °C for Fe_2_CoGa, and then held there for 10 h to ensure homogeneity, and then slowly cooled to 900 °C. The single crystallinity was checked by white‐beam backscattering Laue XRD (Bruker D8 VENTURE) at room temperature. All samples showed sharp and well‐defined Laue spots that can be indexed with a single pattern, indicating the high quality of the as‐grown crystals. Powder XRD measurements were performed with Co K*α*1 radiation (*λ* = 1.788965 Å) on powders obtained by grinding the SC. Quantitative electron probe microanalysis of the crystals was performed using a WDX spectrometer (Cameca SX 100) using the pure elements as standards. ICP‐OES analysis was performed using an Agilent 5100 SVDV ICP‐OES. The matrix‐matched standards for the calibration of the spectrometer were prepared from single‐element standards. Then, the ^57^Fe Mössbauer spectra of Fe_2_ *YZ* Heusler phases were measured at room temperature with a standard WissEl spectrometer which was operated in the constant acceleration mode and equipped with a ^57^Co/Rh source. Powdered samples containing approximately 10 mg cm^−2^ of Fe were obtained by intensive grinding of the SC, which were then diluted with boron nitride to ensure homogeneous distribution in acrylic glass sample containers. The data were evaluated with the MossWinn^[^ ^54^ ^]^ program using the thin absorber approximation. Gaussian hyperfine field distributions were used to decompose the magnetic hyperfine patterns.

### Magnetic and Electrical Transport Measurements

Magnetization measurements were performed using an MPMS Quantum Design vibrating sample magnetometer. The electrical transport properties were characterized by a Quantum Design physical property measurement system (PPMS) using the ACT option. A standard four‐probe method was used for all measurements. To correct for contact misalignment, the measured data were field symmetrized and antisymmetrized for longitudinal resistivity and Hall resistivity, respectively.

### Thermoelectric and Thermal Transport Measurements

All thermal transport experiments were performed using the PPMS with the one‐heater two thermometer configuration. The Seebeck thermopower and thermal conductivity were measured using the thermal transport option (TTO) of the PPMS. The Nernst thermopower under a magnetic field was measured using the PPMS, an external nanovoltmeter, and a current source (Keithley) controlled by LabVIEW software. The temperature gradient was generated using a resistive heater, connected to a gold‐coated flat copper wire at one end of the sample. The thermal gradient Δ*T* was applied along the [100] direction of the crystal, while the magnetic field was applied along the [110] direction. The crystal was attached to a heat sink using another flat copper wire. For temperature gradient (Δ*T*) measurements, two gold‐plated copper leads were attached directly to the crystal using the silver epoxy. The distance between the thermometers was 2–3 mm. The Δ*T* was typically set to 1–3% of the base temperature. Two copper wires were attached to the transverse direction of the crystal using the silver epoxy to measure the Nernst voltage. The Seebeck thermopower was estimated using the relation *S*_xx_ = *V*_x_/Δ*T*_x_, where *V*_x_ is the longitudinal voltage. The Nernst thermopower was estimated as *S*_xy_ = *L*_x_ *V*_y_/(*L*_y_Δ*T*_x_), where *V*_y_ is the transverse voltage, *L*_x_ and *L*_y_ are the distance between two temperature leads, and the distance between two voltage wires, respectively. To correct the data for contact misalignment, the measured data were field antisymmetrized.

### DFT Calculations

The theoretical investigations were conducted by employing ab initio calculations based on DFT as implemented in VASP.^[^ ^55^ ^]^ In this code, plane waves and pseudopotentials were used as a basis set, and the exchange‐correlation potential was taken as the generalized gradient approximation (GGA).^[^ ^56^ ^]^ The k mesh used for the integration over the Brillouin zone was 13 × 13 × 13. In the next step, Wannier functions were extracted from the DFT results using the Wannier90 package.^[^ ^57^ ^]^ From these Wannier functions, a Tight‐Binding Hamiltonian H was constructed and used to evaluate the BC Ω in the system as:^[^ ^18^, ^58^, ^59^ ^]^

(1) $$\Omega \;=\;\sum_{m\neq n}\frac{\langle n\left|\frac{\partial H}{\partial k_{i}}\right|m\rangle \langle m\left|\frac{\partial H}{\partial k_{j}}\right|n\rangle -\left(i↔j\right)}{{\left(E_{n}-E_{m}\right)}^{2}}$$

where |*n*〉 and *E_n_* are eigenstates and eigenenergies of H. From this, the anomalous Hall conductivity is calculated as^[^ ^18^, ^58^ ^]^

(2) $$\sigma_{\mathrm{xy}}=\frac{e^{2}}{\hbar }\;\sum_{n}\int \frac{d^{3}k}{{\left(2\pi \right)}^{3}}\Omega_{\mathrm{xy}}^{z}f_{n}$$

and the anomalous Nernst conductivity as^[^ ^58^, ^60^ ^]^

(3) $$\alpha_{\mathrm{xy}}=\;-\frac{1}{T}\frac{e}{\hbar }\sum_{n}\int \frac{d^{3}k}{{\left(2\pi \right)}^{3}}\Omega_{\mathrm{xy}}^{z}\left[\left(E_{n}-E_{F}\right)f_{n}+k_{B}T\ln\left(1+e^{\frac{E_{n}-E_{F}}{-k_{B}T}}\right)\right]$$

where *f_n_* is the Fermi distribution function, *T* is the actual temperature, and *E*_F_ is the Fermi level. The k mesh for the integration over the Brillouin zone in this step was chosen as 301 × 301 × 301 to ensure converged results. The calculated *σ*_xy_ used in Figure 2c were obtained from the Figures S6c–S13c, Supporting Information, by reading off the values at *E* − *E*_F_ = 0. The *σ*_xy_ and *α*_xy_ were calculated at 0 K and 300 K, respectively.

## Conflict of Interest

The authors declare no conflict of interest.

## Supporting information

Supporting Information

## Data Availability

The data that support the findings of this study are available from the corresponding author upon reasonable request.
