# Supplementary material for: Large Anomalous Hall and Nernst Effects in High Curie‐Temperature Iron‐Based Heusler Compounds
Source: Adv Sci (Weinh). 2021 Jul 8;8(17):2100782. doi: 10.1002/advs.202100782 (PMC8425906; doi:10.1002/advs.202100782)
Supplement: Supplementary file 1 — Supporting Information [file ADVS-8-2100782-s001.pdf]

WILEY-VCH

© 2021 Wiley-VCH GmbH

ADVANCED SCIENCE
Open Access

Supporting Information

for *Adv. Sci.,* DOI: 10.1002/advs.202100782

Large Anomalous Hall and Nernst effects in High Curie-temperature Iron-based Heusler Compounds

Felix Mende, Jonathan Noky, Satya N. Guin, Gerhard H. Fecher, Kaustuv Manna, Peter Adler, Walter Schnelle, Yan Sun, Chenguang Fu, Claudia Felser**

© 2021 Wiley-VCH GmbH

Supporting Information

Large Anomalous Hall and Nernst effects in High Curie-temperature Iron-based Heusler Compounds

Felix Mende, Jonathan Noky, Satya N. Guin, Gerhard H. Fecher, Kaustuv Manna, Peter Adler, Walter Schnelle, Yan Sun, Chenguang Fu, Claudia Felser**

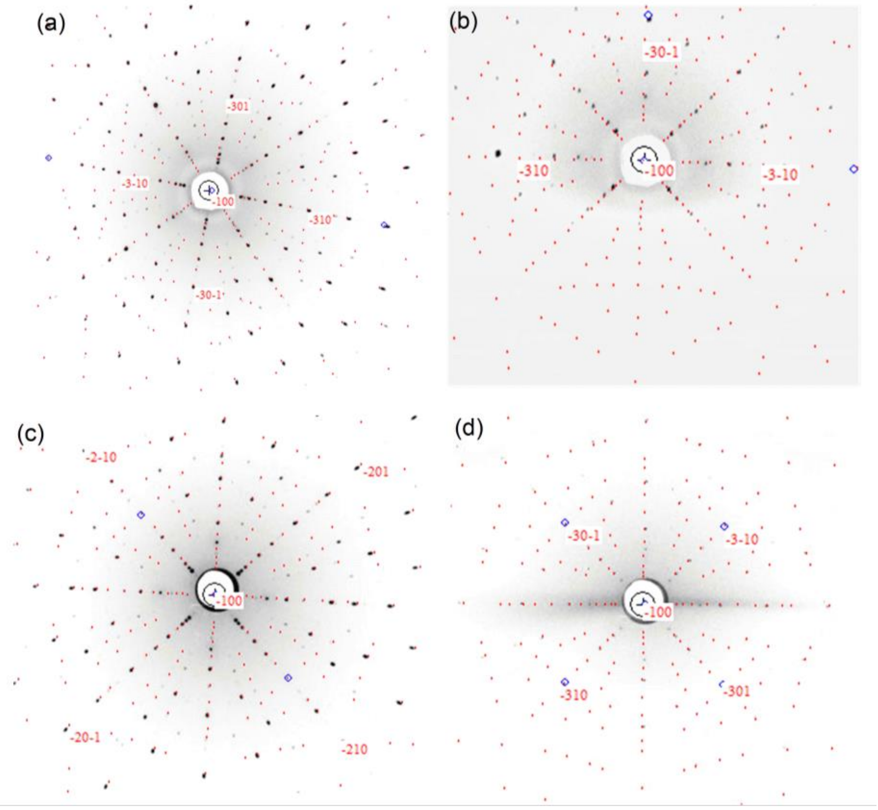

Figure S1: Laue diffraction pattern of the oriented single crystals of (a) Fe_2CoAl, (b) Fe_2CoGa, (c) Fe_2NiAl, (d) Fe_2NiGa. The black spots are the observed pattern. And the red spots are the calculated pattern along with Miller indices.

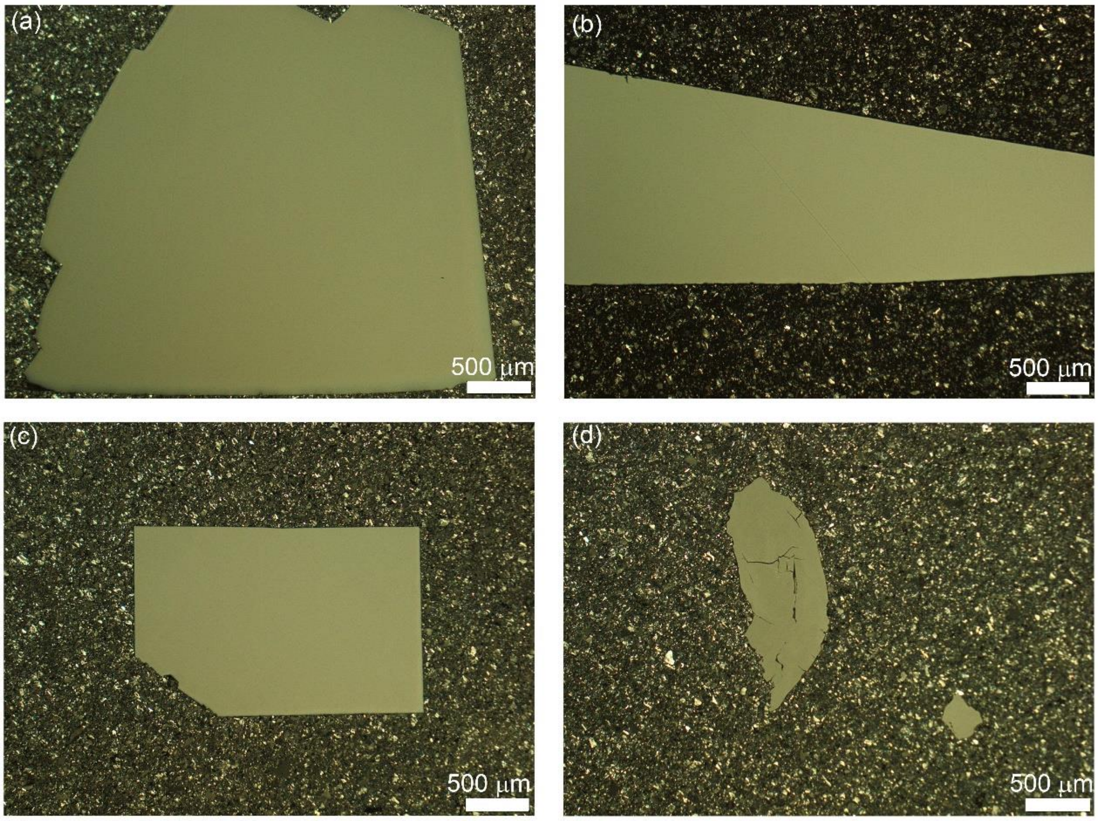

Figure S1: The polarized light microscopy images of (a) Fe_2CoAl, (b) Fe_2CoGa, (c) Fe_2NiAl, (d) Fe_2NiGa. The images indicate the high homogeneity of the single crystal pieces.

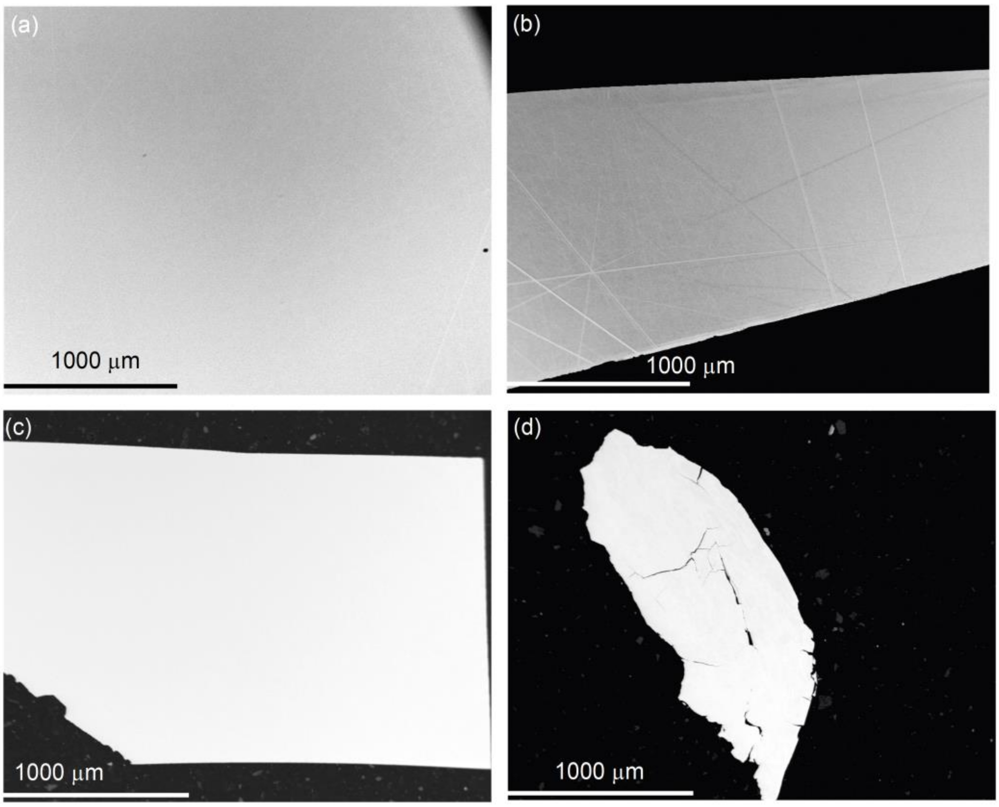

Figure S2: backscattered electron (BSE) microscopy images of (a) Fe_2CoAl, (b) Fe_2CoGa, (c) Fe_2NiAl, (d) Fe_2NiGa. The BSE images indicate the high homogeneity of the single crystal pieces.

Chemical composition

The chemical composition of the single-crystalline compounds was double checked with WDX and ICP-OES analysis. For WDX the embedded and polished single crystalline pieces were measured at 10 different spots. The final composition was obtained by averaging over these 10 positions.

Table S1. Averaged WDX analysis for Fe_2YZ single crystals.

	Element	mass %	Deviation mass%	Stoichiometric ratio
Fe_2CoAl	Fe	49.66	0.17	1.9860 ± 0.007
	Co	25.47	0.09	1.0188 ± 0.004
	Al	24.86	0.13	0.9940 ± 0.005
Fe_2CoGa	Fe	54.83	0.16	2.193 ± 0.006
	Co	24.67	0.04	0.987 ± 0.002
	Ga	20.50	0.13	0.820 ± 0.005
Fe_2NiAl	Fe	56.87	0.23	1.987±0.008
	Ni	29.55	0.12	0.983±0.008
	Al	14.24	0.03	1.023±0.004
Fe_2NiGa	Fe	50.85	0.13	2.034±0.005
	Ni	24.5	0.07	0.980±0.003
	Ga	24.65	0.08	0.986±0.003

Table S2. ICP-OES analysis for Fe_2YZ single crystals.

	Element	mass %	Deviation mass%	stoichiometric ratio
Fe_2CoAl	Fe	50.98	0.21	1.994 ± 0.009
	Co	27.56	0.14	1.022 ± 0.011
	Al	12.16	0.05	0.984 ± 0.009
Fe_2CoGa	Fe	49.09	0.25	2.167 ± 0.012
	Co	23.68	0.2	0.991 ± 0.021
	Ga	23.84	0.08	0.843 ± 0.008
Fe_2NiAl	Fe	51.38	0.14	2.055±0.005
	Ni	22.78	0.05	0.911±0.02
	Al	25.84	0.11	1.033±0.004
Fe_2NiGa	Fe	47.93	0.14	2.047±0.003
	Ni	24.32	0.11	0.988±0.005
	Ga	28.18	0.19	0.964±0.007

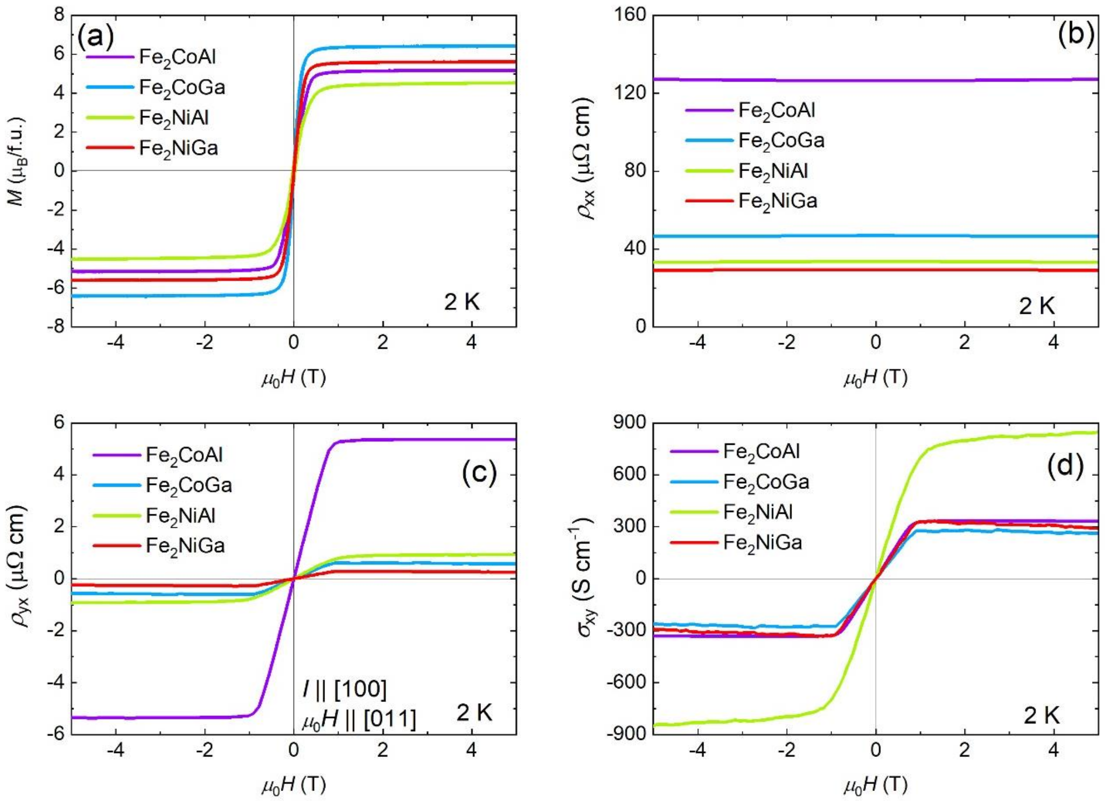

Figure S4. Magnetic and electrical properties of the as-grown Fe_2YZ single crystals at 2 K. (a) The magnetic-field-dependent magnetization. (b) Magneto-resistivity. (c) Hall resistivity ρ_{yx} versus magnetic field. (d) Magnetic-field-dependent Hall conductivity σ_{xy}.

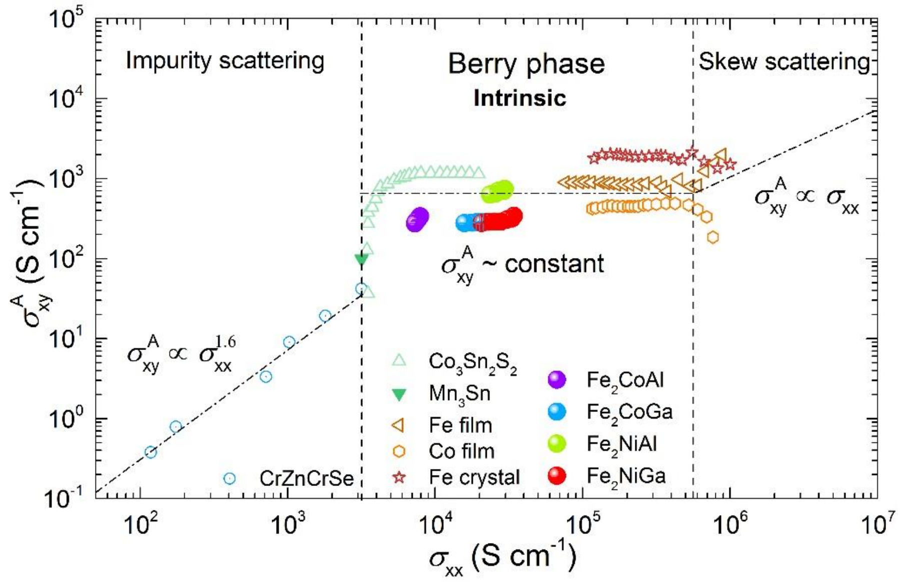

Figure S5: σ_{xy}^{A} versus σ_{xx} in the framework of the unified model.[S1] The data were adapted from the reference (Fig. S12).[S2] The studied Fe_2YZ single crystals locate in the intrinsic region dominated by Berry-phase curvature.

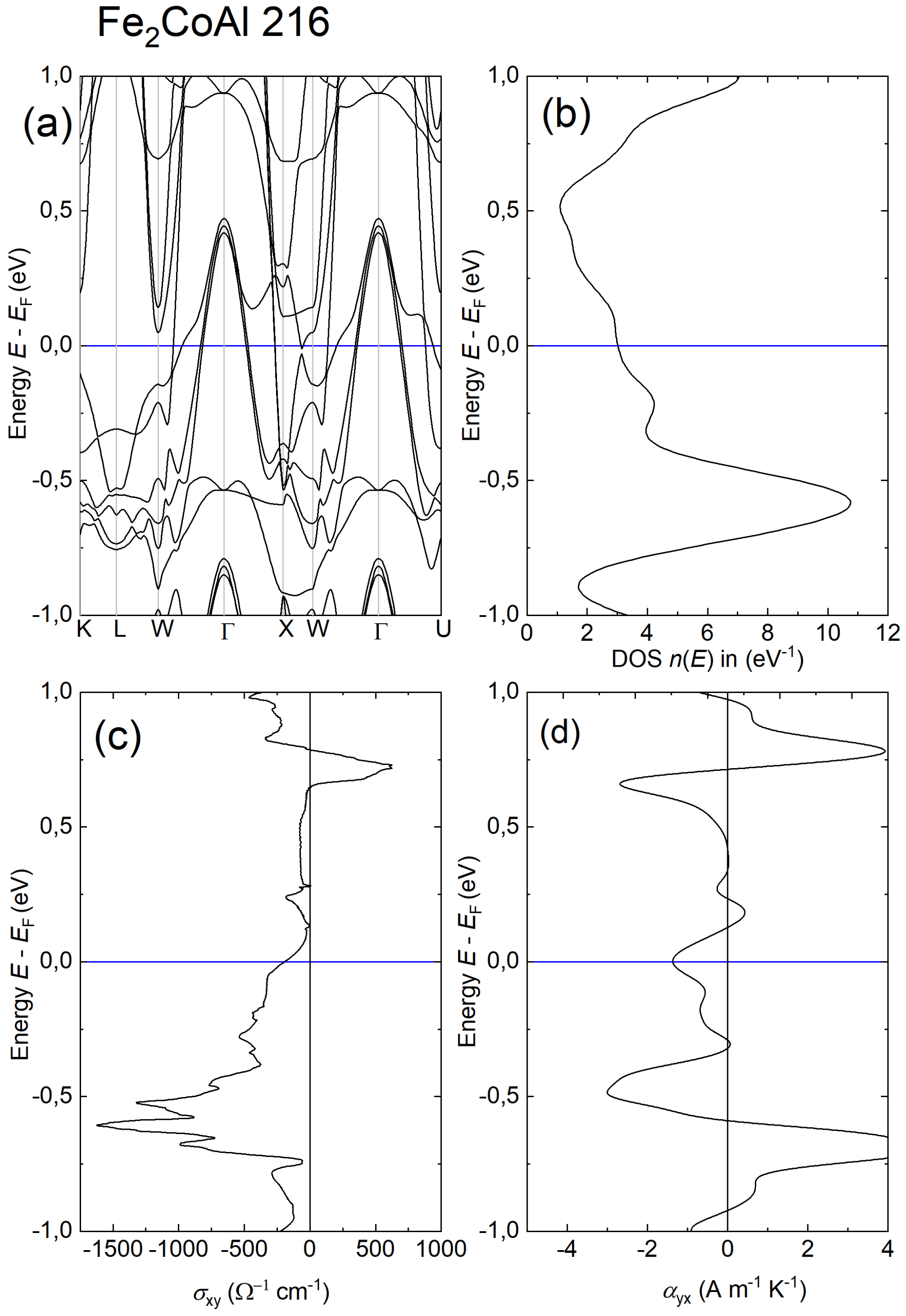

Figure S6: (a) Electronic structure, (b) density of states DOS, (c) calculated anomalous Hall conductivity σ_{xy}, and (d) anomalous Nernst conductivity α_{yx} for Fe_2CoAl in inverse Heusler structure (SG 216) .

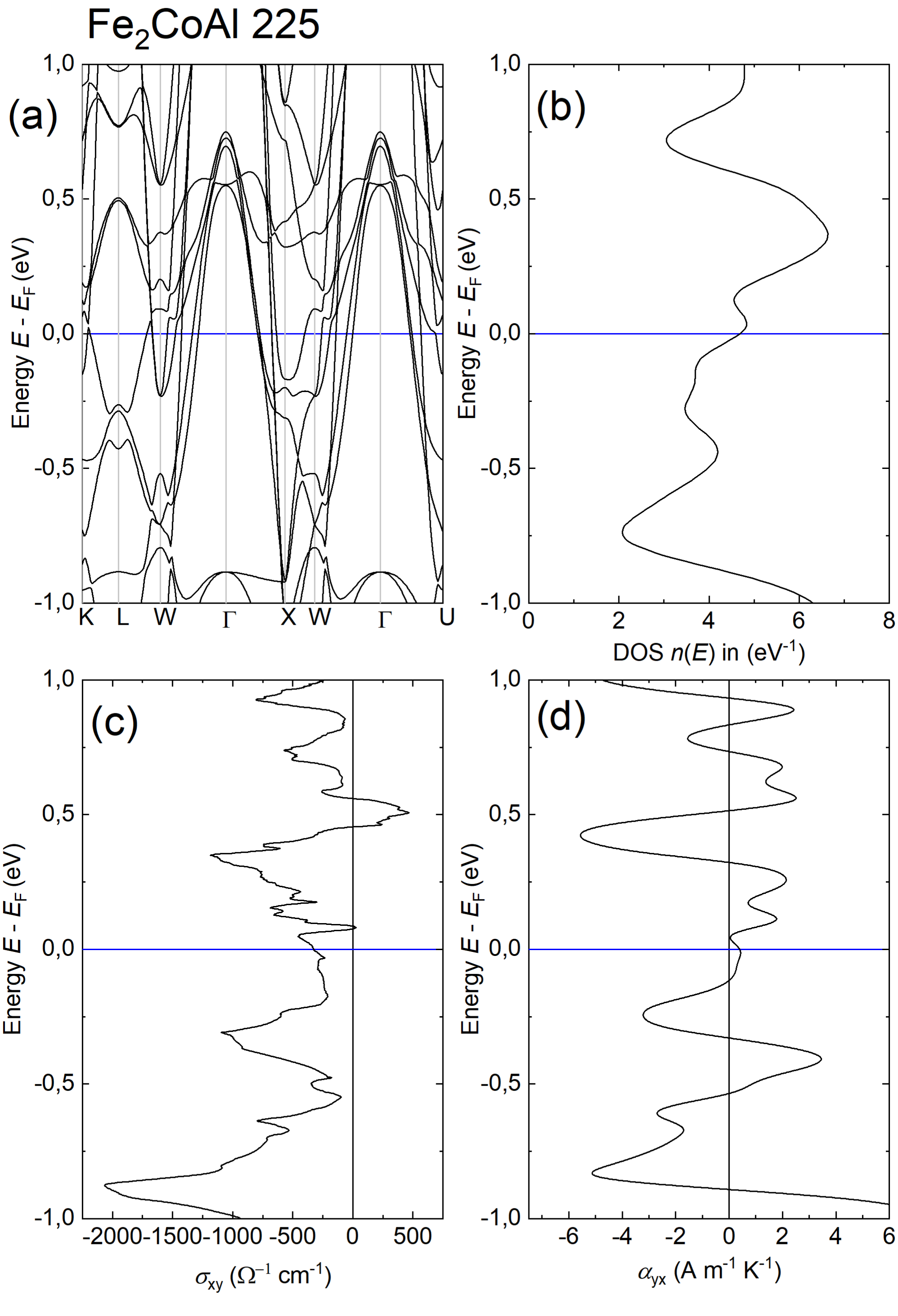

Figure S7: (a) Electronic structure, (b) density of states DOS, (c) calculated anomalous Hall conductivity σ_{xy}, and (d) anomalous Nernst conductivity α_{yx} for Fe_2CoAl in Heusler structure (SG 225).

(a)

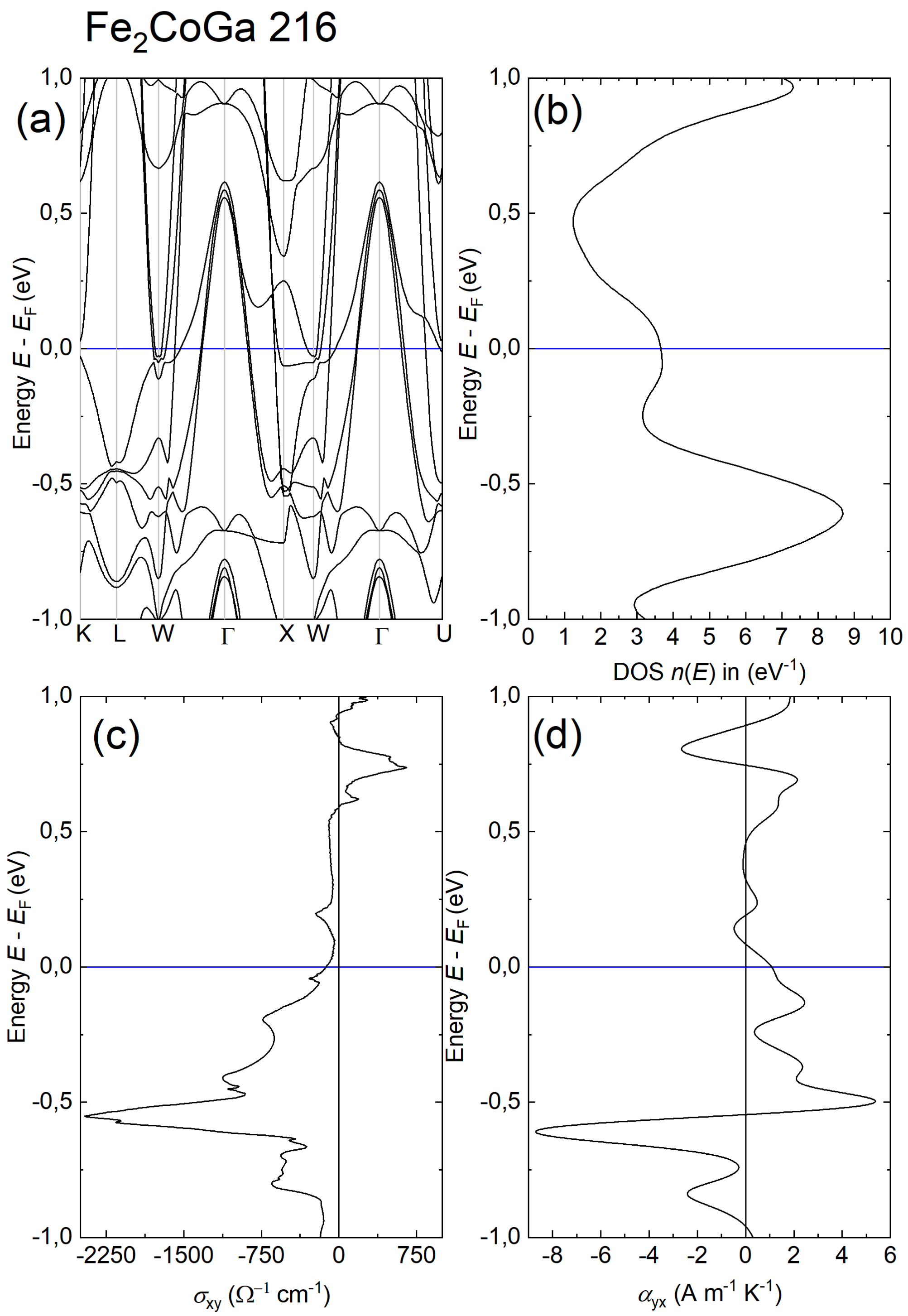

Figure S8: (a) Electronic structure, (b) density of states DOS, (c) calculated anomalous Hall conductivity σ_{xy}, and (d) anomalous Nernst conductivity α_{yx} for Fe_2CoGa in inverse Heusler structure (SG 216).

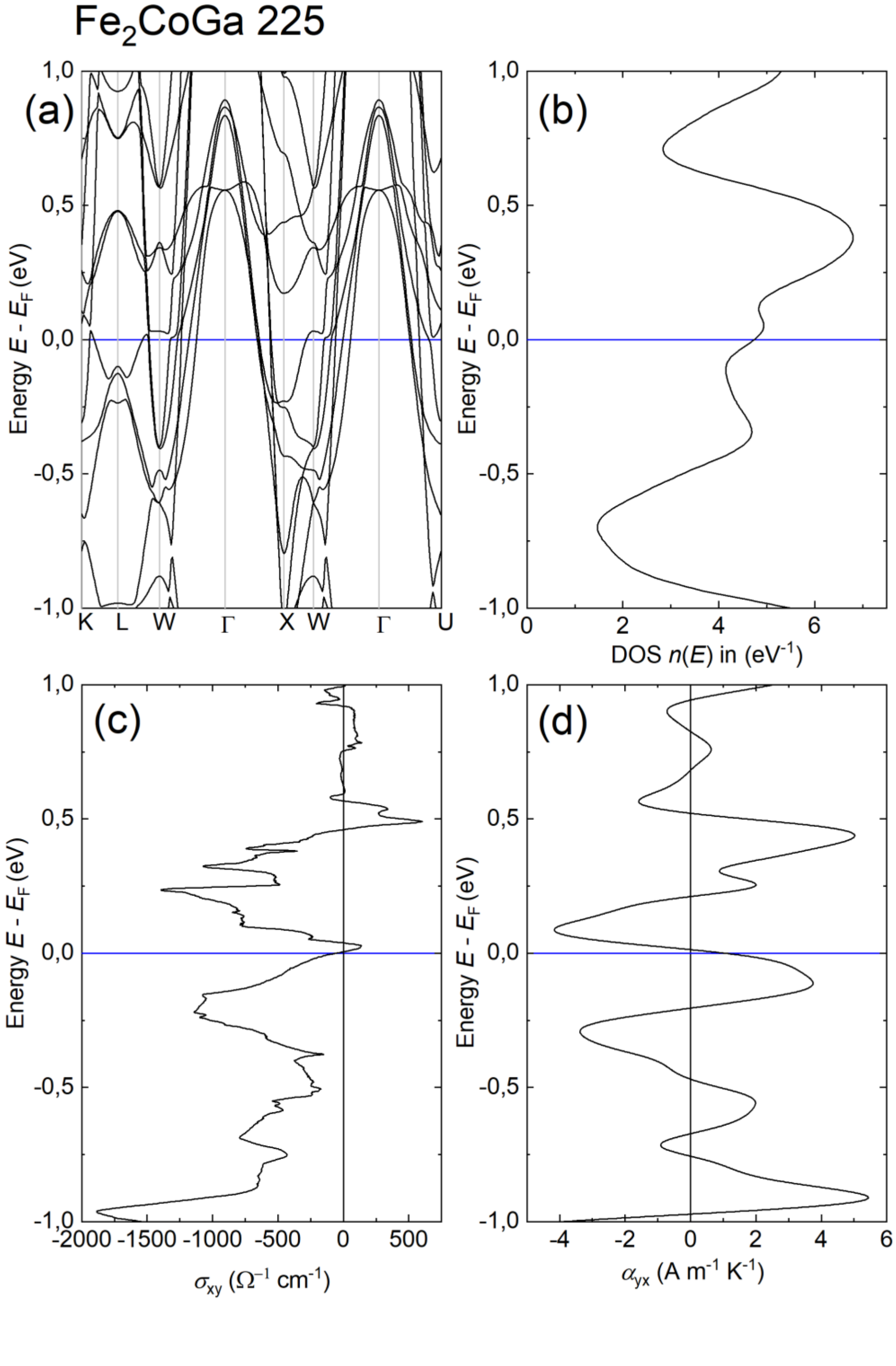

Figure S9: (a) Electronic structure, (b) density of states DOS, (c) calculated anomalous Hall conductivity σ_{xy}, and (d) anomalous Nernst conductivity α_{yx} for Fe_2CoGa in Heusler structure (SG 225).

(b)

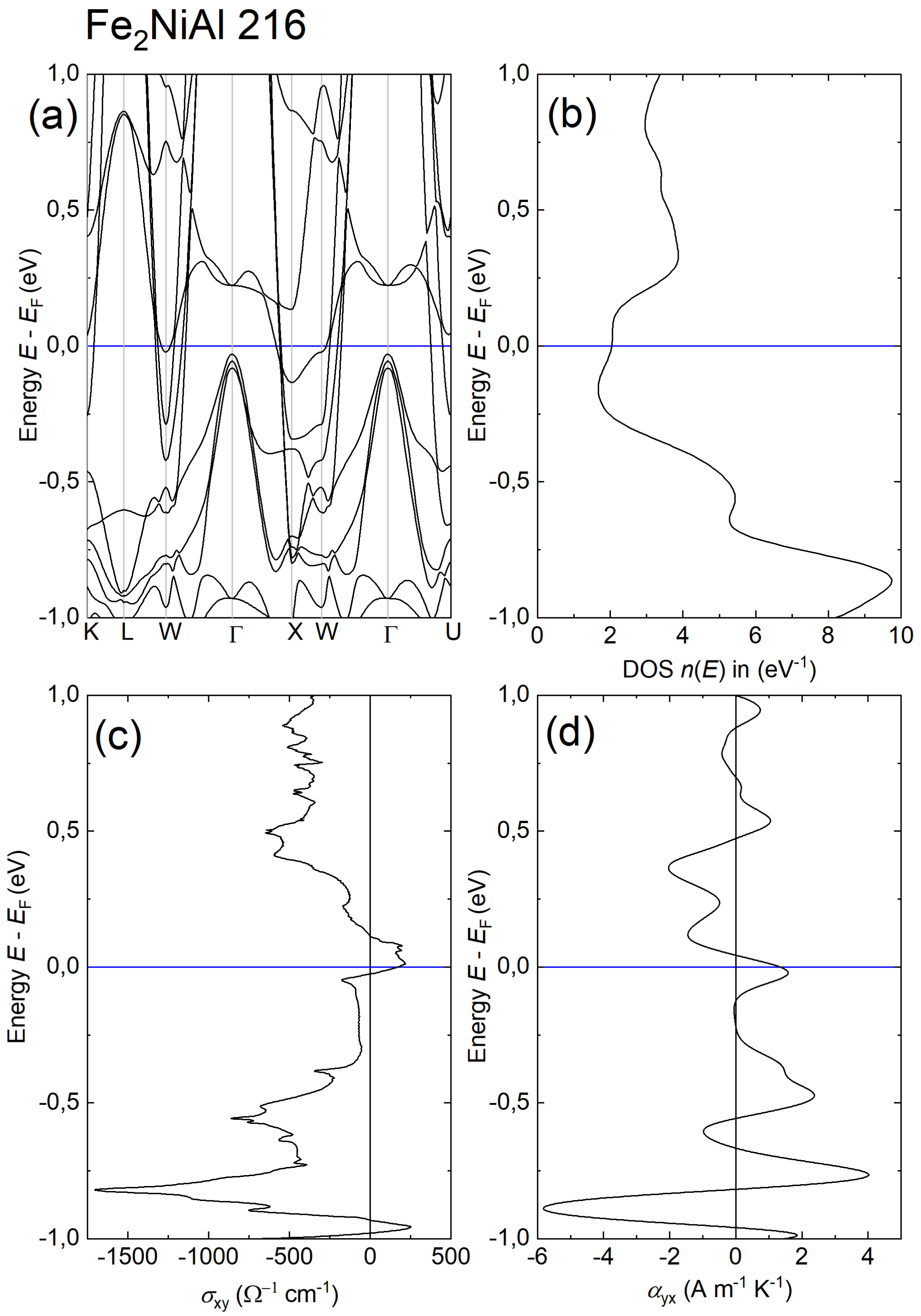

Figure S10: (a) Electronic structure, (b) density of states DOS, (c) calculated anomalous Hall conductivity σ_{xy}, and (d) anomalous Nernst conductivity α_{yx} for Fe_2NiAl in inverse Heusler structure (SG 216).

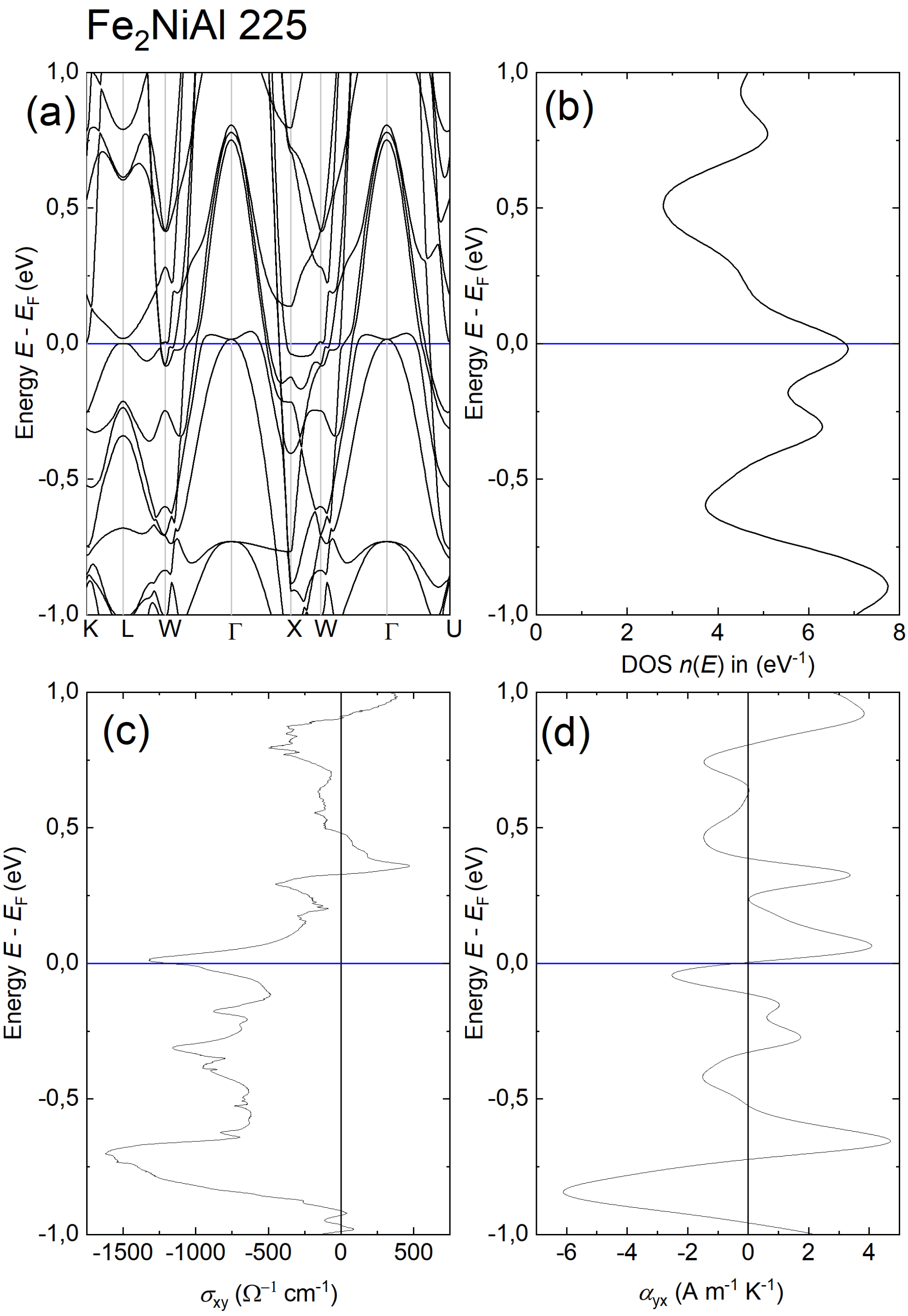

Figure S11: (a) Electronic structure, (b) density of states DOS, (c) calculated anomalous Hall conductivity σ_{xy}, and (d) anomalous Nernst conductivity α_{yx} for Fe_2NiAl in Heusler structure (SG 225).

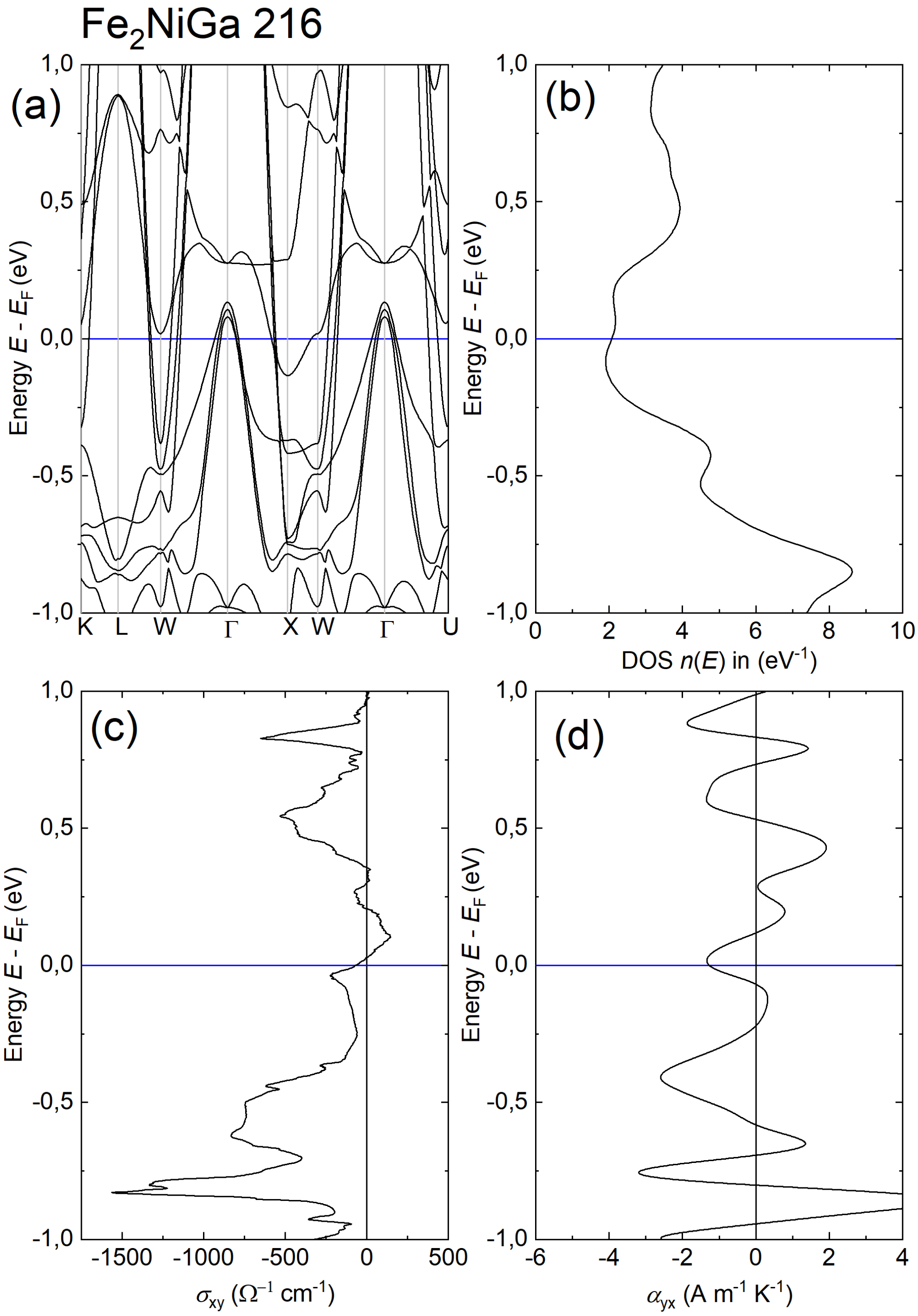

Figure S12: (a) Electronic structure, (b) density of states DOS, (c) calculated anomalous Hall conductivity σ_{xy}, and (d) anomalous Nernst conductivity α_{yx} for Fe_2NiGa in inverse Heusler structure (SG 216).

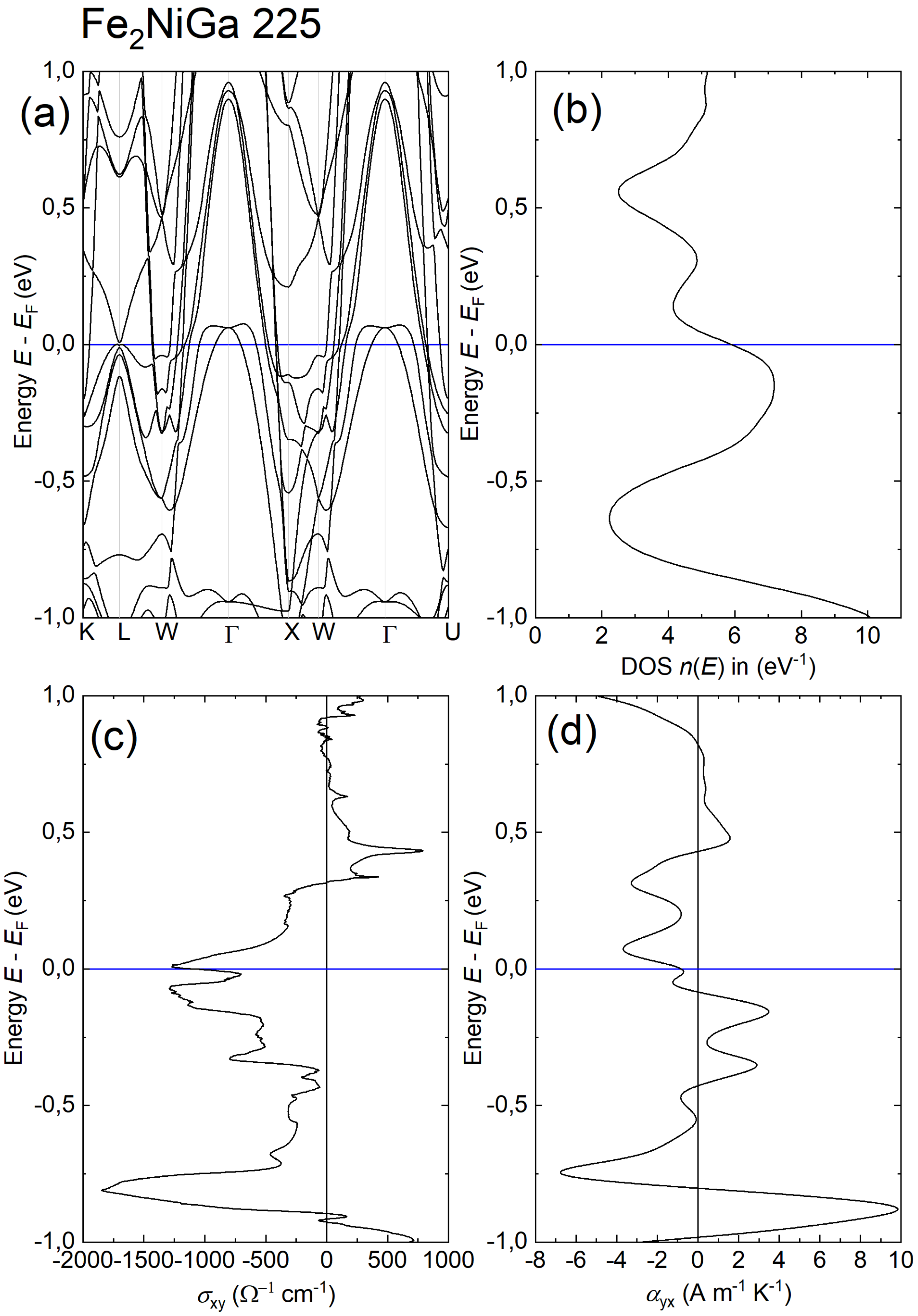

Figure S13: (a) Electronic structure, (b) density of states DOS, (c) calculated anomalous Hall conductivity σ_{xy}, and (d) anomalous Nernst conductivity α_{yx} for Fe_2NiGa in Heusler structure (SG 225).

The judgment of disorder type

For inverse Heusler compounds (SG 216), we can define the Wyckoff positions 4*a* (0, 0, 0), 4*b* (1/2, 1/2, 1/2), 4*c* (1/4, 1/4, 1/4), 4*d* (3/4, 3/4, 3/4), where for Fe_2YZ compounds one Fe and the *Y* atom (*Y* = Co, Ni) are located on 4*a* and 4*b* sites, while the other Fe atom and the Z atom (Z = Al, Ga) reside on 4*d* and 4*c* sites, respectively. In the regular Heusler structure (SG 225) both, 4*a* and 4*b* sites would be occupied by Fe atoms. Important structure factors for determination of the disorder are then:

$F(111) = |4[(f_{4a}-f_{4b})^2 + (f_{4c}-f_{4d})^2]^{1/2}|$

$F(200) = |4(f_{4a}-f_{4c}+f_{4b}-f_{4d})|$

$F(220) = 2|4(f_{4a}+f_{4b}+f_{4c}+f_{4d})|$

Here f_{4a}, f_{4b}, $f_{4c,}$ and f_{4d} are the averaged atom form factors of the atoms at positions 4a, 4b, 4c, and 4d.[S3]

According to the above equations, if the positions 4a and 4b, as well as 4c and 4d, have the same averaged atom form factor, F(111) will be zero and the (111) reflexion will vanish, indicating B2-type disorder (SG 221). Moreover, if all the positions 4a, 4b, 4c, and 4d have the same averaged atom form factor, both (111) and (200) reflexions will vanish, indicating an A2-type disorder (SG 229).

The powder XRD patterns for the different site occupancies were simulated. As shown in Table S3, the low indexed reflexions (111) and (200) are directly related to the crystal structure and site occupations. They are well defined for Fe_2CoAl but become very tiny in the case of Fe_2CoGa due to the similar scattering factors of Ga and the 3d elements. The reflexions of Fe_2NiAl and Fe_2NiGa will behave in the same way. It is obvious from the intensities that the ordered X and $L2_1$ structures exhibit clear (111) and (200) diffraction peaks. The (111) reflexion vanishes for B2 type disordered structure.

Table S3. XRD intensities for ordered and disordered Fe_2CoZ.

	Fe_2CoAl		Fe_2CoGa	
Structure type	I_{111} / I_{220}	I_{200} / I_{220}	I_{111} / I_{220}	I_{200} / I_{220}
X	6.88%	4.14%	0.84%	0.26%
$L2_1$	8.12%	2.85%	0.49%	0.63%
$D0_3$	7.24%	3.68%	0.68%	0.37%
B2a	0	2.85%	0	0.63%
B2b	0	4.14%	0	0.26%
A2	0	0	0	0

Mössbauer spectra

Room-temperature Mössbauer spectra of Fe_2YZ samples are shown in Figure S17. The spectra indicate a strong degree of atomic disorder for all the samples, while the detailed disordering patterns seem to vary. One can expect that Fe_2CoZ, as well as Fe2NiZ phases, adopt the inverse Heusler structure with one Fe atom residing on 4*d* and the other on 4*a,b* sites. In the case of well-ordered inverse Heusler phases, distinct hyperfine components with typical hyperfine field values (B_{hf}) between 31 and 33 T for the 4*d* and ~20 T for the 4*a,b* sites are found[S4]. For a stoichiometric Fe_2YZ phase the area ratio between the two components should be about 1:1. Pure B2-type interchange between Fe 4*d* and Z 4*c* sites does not change the nearest neighbor local environment of the Fe atoms and thus the shape of the patterns is only affected to a minor extent (line broadening of the 30 T component)[S5]. It has been verified by Mössbauer spectroscopy that Fe_2CoSi[S6] and Fe_2CoGe[S7] adopt the inverse Heusler structure.

Pronounced hyperfine components with B_{hf} larger than 30 T in the spectra of the present Fe_2CoGa, Fe_2CoAl, and Fe_2NiAl samples (Figure S17) indicate Fe atoms sitting on 4*d* sites which suggests that these compounds basically are inverse Heusler phases as expected. In none of the spectra, however, a well-defined second component with B_{hf} ~ 20 T can be identified. Instead, broad features are observed and accounted for by a second component with broad B_{hf} distributions. These observations suggest that the present samples not only feature B2-type disorder, but also considerable disorder between the Fe 4*a,b*, and *Z* 4*c* sites which is in agreement with the enhanced fraction of Fe components with B_{hf} values near 30 T.

For the various Fe_2YZ phases studied here Mössbauer spectra have been reported previously. Thus, annealed samples of Fe_2CoGa revealed Mössbauer spectra with two sharp components having B_{hf} values of 31 and 23.5 T[S8], in agreement with the expectations for an inverse Heusler structure. The spectrum of the present sample still shows some structure but the broad second component with enhanced average B_{hf} suggests an increased level of atomic disorder. The spectral shape of the Fe_2CoAl sample is similar to that reported previously[S9] and also indicates strong disorder. Remarkably, the spectrum of Fe_2NiAl is somewhat sharper and the second broader component is characterized by a high average B_{hf} of 31 T, whereas the other component has B_{hf} ~33 T. This could indicate that either the fraction of Fe atoms on the 4*a,b* sites is small or that the B_{hf} of Fe atoms at the 4*a,b* sites is enhanced. In addition to the magnetic hyperfine pattern, the Fe_2NiAl spectrum shows a broad single line corresponding to a paramagnetic fraction which was also apparent in the Mössbauer spectra of an earlier sample of Fe_2NiAl[S9]. Finally, the spectrum of Fe_2NiGa differs from the others as it is even broader and has a decreased average B_{hf}. No distinct signal with B_{hf} > 30 T is observed. The

broad spectrum is in agreement with earlier results[S7] and it was suggested that in Fe_2NiGa strong mixing between all the sites leads to an A2-like disorder.

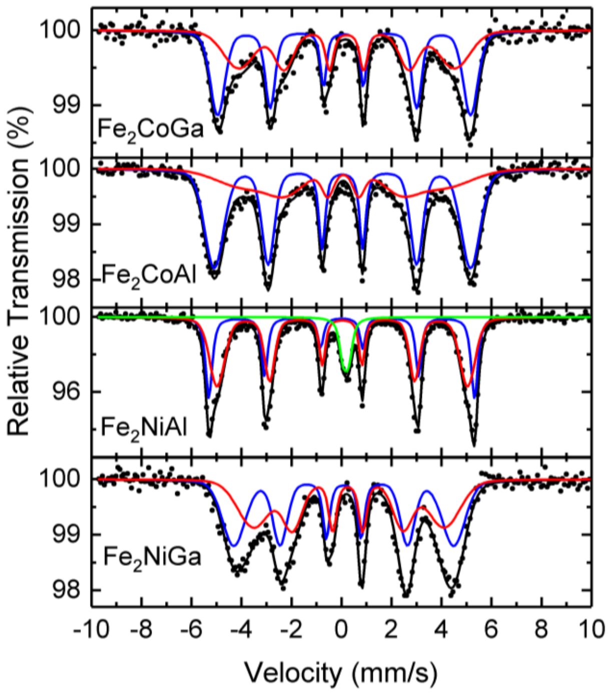

Figure S14: Mössbauer spectra of Fe_2YZ Heusler compounds. Dots correspond to the experimental data, black and colored lines to the calculated total and subspectra, respectively.

DSC measurements

Before doing the Bridgman growth of single crystals, DSC measurements were first performed on polycrystalline Fe_2YZ samples using Al_2O_3 crucibles with caps to determine the melting and solidification peaks (Figure S18). During some of the measurements (Fe_2CoAl and Fe_2NiAl), the caps dropped when the temperature reaches near the order-disorder transition point (*T*m) of the samples, affecting the detection of *T*m. To confirm the *T*m, further DSC measurements were performed using the as-grown single crystals, as presented in Figure S19. *T*m is clearly observed in both heating and cooling DSC curves.

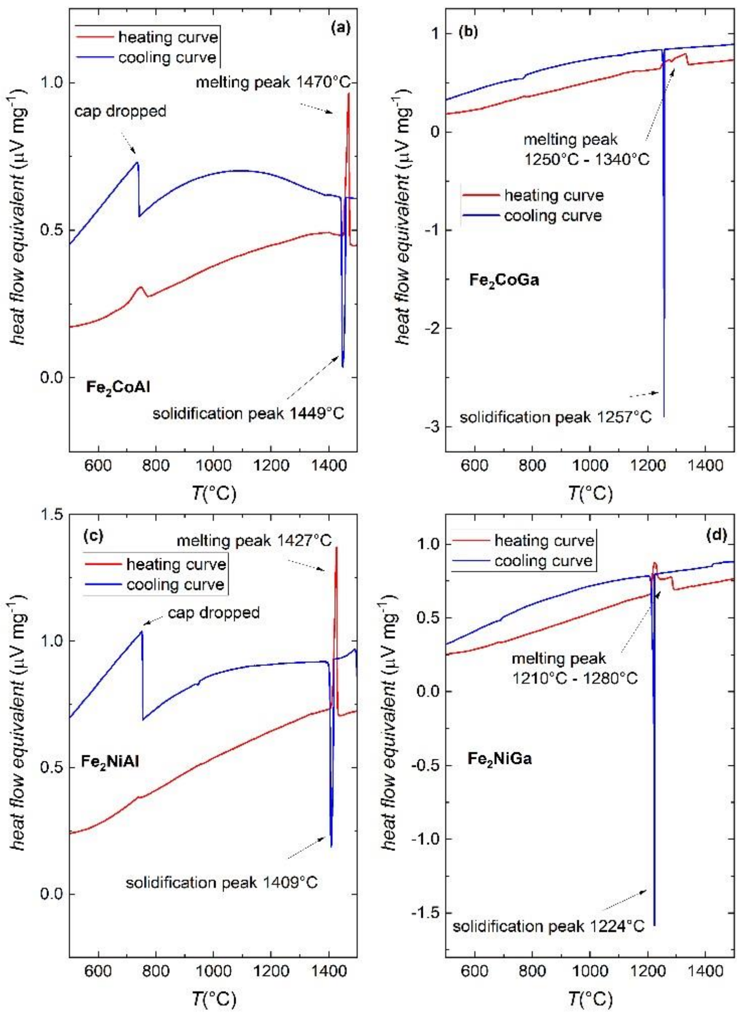

Figure S15: Heat and cooling DSC curves measured using Fe_2YZ polycrystalline samples after arc-melting and annealing. The melting and solidification peaks (regions) are revealed.

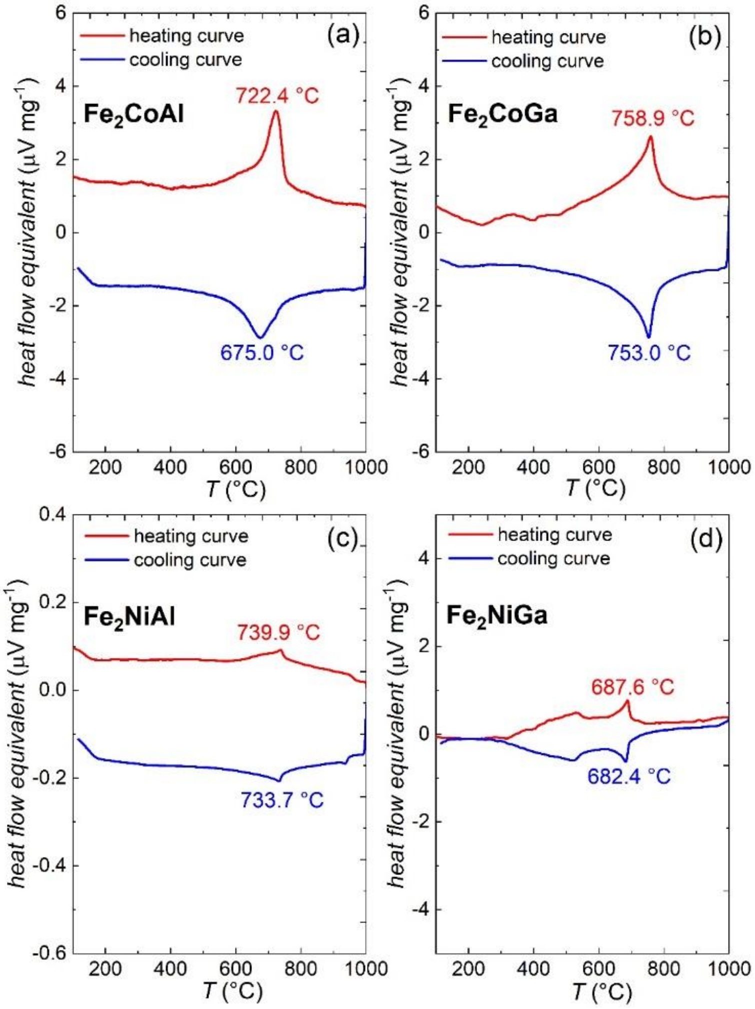

Figure S16: Heat and cooling DSC curves measured using the as-grown Fe_2YZ single crystals. The order-disorder transition is observed.

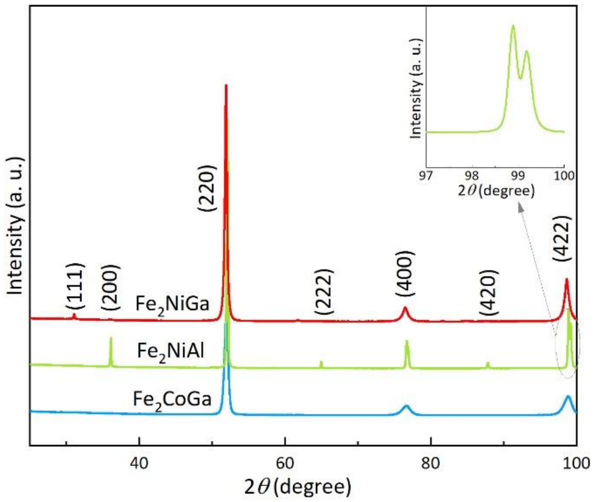

Figure S17. Powders XRD pattern for the Fe_2YZ single crystals after annealing.

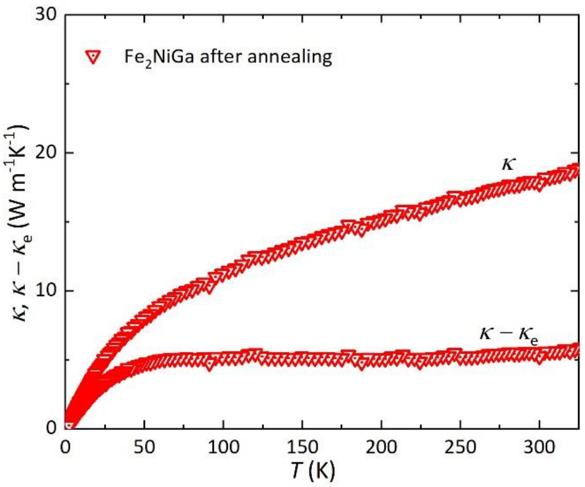

Figure S18. Temperature dependence of thermal conductivity κ for t Fe_2NiGa after annealing.

References:

[S1] a) S. Onoda, N. Sugimoto, N. Nagaosa, *Phys. Rev. Lett.* **2006**, *97*, 126602; b) T. Miyasato, N. Abe, T. Fujii, A. Asamitsu, S. Onoda, Y. Onose, N. Nagaosa, Y. Tokura, *Phys. Rev. Lett.* **2007**, *99*, 086602.

[S2] E. Liu, Y. Sun, N. Kumar, L. Muechler, A. Sun, L. Jiao, S.-Y. Yang, D. Liu, A. Liang, Q. Xu, J. Kroder, V. Süß, H. Borrmann, C. Shekhar, Z. Wang, C. Xi, W. Wang, W. Schnelle, S. Wirth, Y. Chen, S. T. B. Goennenwein, C. Felser, *Nat. Phys.* **2018**, *14*, 1125.

[S3] P. J. Webster, *Contemp. Phys.*, **1969**, *10*, 559.

[S4] V. Jung, B. Balke, G. H. Fecher, C. Felser, *Appl. Phys. Lett.* **2008**, *93*, 042507.

[S5] J. Karel, J. E. Fischer, S. Fabbrici, E. Pippel, P. Werner, M. V. Castergnaro, P. Adler, S. Ouardi, B. Balke, G. H. Fecher, J. Morais, F. Albertini, S. S. P. Parkin, and C. Felser, *J. Mater. Chem. C* **2017**, *5*, 4388.

[S6] J. E. Fischer, J. Karel, S. Fabbrici, P. Adler, S. Ouardi, G. H. Fecher, F. Albertini, C. Felser, *Phys. Rev. B* **2016**, *94*, 024418.

[S7] T. Gasi, V. Ksenofontov, J. Kiss, S. Chadov, A. K. Nayak, M. Nicklas, J. Winterlik, M. Schwall, P. Klaer, P. Adler, C. Felser, *Phys. Rev. B* **2013**, *87*, 064411.

[S8] N. K. Jaggi, K. R. P. M. Rao, A. K. Grover, L. C. Gupta, R. Vijayaraghavan, L. D. Khoi, *Hyp. Intact.* **1978**, *4*, 402.

[S9] E. S. Popiel, W. Zarek, M. Tuszyński, Nukleonika **2004**, *49* (Suppl. 3), S49.
